# Random Codon Re-encoding Induces Stable Reduction of Replicative Fitness of Chikungunya Virus in Primate and Mosquito Cells

**DOI:** 10.1371/journal.ppat.1003172

**Published:** 2013-02-21

**Authors:** Antoine Nougairede, Lauriane De Fabritus, Fabien Aubry, Ernest A. Gould, Edward C. Holmes, Xavier de Lamballerie

**Affiliations:** 1 Aix Marseille Univ, IRD French Institute of Research for Development, EHESP French School of Public Health, UMR_D 190 “Emergence des Pathologies Virales,” Marseille, France; 2 Sydney Emerging Infections and Biosecurity Institute, School of Biological Sciences and Sydney Medical School, The University of Sydney, Sydney, New South Wales, Australia; 3 Fogarty International Center, National Institutes of Health, Bethesda, Maryland, United States of America; Institut Pasteur, France

## Abstract

Large-scale codon re-encoding represents a powerful method of attenuating viruses to generate safe and cost-effective vaccines. In contrast to specific approaches of codon re-encoding which modify genome-scale properties, we evaluated the effects of random codon re-encoding on the re-emerging human pathogen Chikungunya virus (CHIKV), and assessed the stability of the resultant viruses during serial *in cellulo* passage. Using different combinations of three 1.4 kb randomly re-encoded regions located throughout the CHIKV genome six codon re-encoded viruses were obtained. Introducing a large number of slightly deleterious synonymous mutations reduced the replicative fitness of CHIKV in both primate and arthropod cells, demonstrating the impact of synonymous mutations on fitness. Decrease of replicative fitness correlated with the extent of re-encoding, an observation that may assist in the modulation of viral attenuation. The wild-type and two re-encoded viruses were passaged 50 times either in primate or insect cells, or in each cell line alternately. These viruses were analyzed using detailed fitness assays, complete genome sequences and the analysis of intra-population genetic diversity. The response to codon re-encoding and adaptation to culture conditions occurred simultaneously, resulting in significant replicative fitness increases for both re-encoded and wild type viruses. Importantly, however, the most re-encoded virus failed to recover its replicative fitness. Evolution of these viruses in response to codon re-encoding was largely characterized by the emergence of both synonymous and non-synonymous mutations, sometimes located in genomic regions other than those involving re-encoding, and multiple convergent and compensatory mutations. However, there was a striking absence of codon reversion (<0.4%). Finally, multiple mutations were rapidly fixed in primate cells, whereas mosquito cells acted as a brake on evolution. In conclusion, random codon re-encoding provides important information on the evolution and genetic stability of CHIKV viruses and could be exploited to develop a safe, live attenuated CHIKV vaccine.

## Author Summary

Emerging arthropod-borne viruses (arboviruses) are a major cause of human and animal morbidity and mortality. Climatic and anthropological activities are responsible for the dispersal of arbovirus transmission vectors into new territories. Chikungunya virus (CHIKV) is an important example of a re-emerging pathogen for which no licensed vaccine exists. One of the vectors of CHIKV, the mosquito *Aedes albopictus*, has dispersed into new temperate regions resulting in outbreaks where they had not been previously observed. Here, we demonstrate that random codon re-encoding, a method that modifies the nucleic acid composition of large coding regions without modifying the encoded proteins, can significantly decrease the replicative fitness of CHIKV. This powerful method of attenuating viruses has several potential advantages for vaccine development, including the possibility to modulate precisely the degree of replicative fitness loss and to generate safe, live-attenuated vaccines that confer long-term protection, in a cost effective manner. Our studies also demonstrate that these re-encoded viruses exhibit a stable phenotype, and that the response to codon re-encoding was largely compensatory in nature, with little reversion of mutations. Finally, we provide further evidence that many synonymous sites in RNA viruses are not neutral and clearly impact viral fitness.

## Introduction

Many emerging infectious diseases are caused by arthropod-borne viruses (arboviruses), almost all of which are single strand RNA viruses. The major outbreaks of dengue fever [1], West Nile encephalitis [2], Chikungunya fever [3], and Rift Valley fever [4] that have occurred in recent decades, each with a significant impact on human health, highlight the urgent need to understand the factors that allow these viruses to invade new territories or adapt to new host or vector species [5]–[6]. Understanding the factors that shape the adaptability of these rapidly evolving infectious agents may provide new opportunities for their eventual control.

Codon usage bias is an important indicator of the evolutionary forces shaping genomes and could arise either through neutral mutational pressure or because specific synonymous codons are selectively advantageous; for example, by increasing the efficiency and/or accuracy of protein expression by maximizing the match to cellular tRNA abundance. Thus, determining the underlying causes of codon bias has become a key topic in evolutionary genetics [7]. RNA viruses often exhibit codon biases that match the nucleotide biases across viral genomes as a whole, suggesting that background mutational pressure is the dominant factor shaping codon choice. However, natural selection may still act at the scale of overall nucleotide composition [8]. Direct selection for specific codon biases has also been documented. For example, in hepatitis A virus rare codons that utilize non-abundant tRNAs are preferred, slowing down the translation process to ensure proper protein folding [9]. The large-scale re-encoding of codon usage in poliovirus, influenza A virus and bacterial virus T7 by reverse genetic methods resulted in virus attenuation [10]–[15], demonstrating that mutations at synonymous sites can indeed have a major impact on viral fitness. To date, all studies of codon re-coding have employed a specific approach such as codon de-optimisation [12], [14], codon pair de-optimisation [10], [13] and increase of CpG/UpA dinucleotide frequency [11]; but all result in a reduction of viral fitness. Currently, there are no studies of codon re-coding in arboviruses. However, studies of these viruses are of special interest because their evolution is strongly constrained by host alternation, such that mutations that may be advantageous in one host type (e.g. mosquitoes or mammals) are deleterious in another [16].

Chikungunya virus (CHIKV; *Togaviridae*; *Alphavirus*) is a small (60–70 nm), enveloped, single-strand positive-sense RNA virus. Its genome of approximately 12 kb contains two open reading frames (ORFs) encoding non-structural and structural proteins, respectively [17]. In Swahili, “Chikungunya” means “bent walker”, reflecting the severe arthralgia associated with CHIKV infections. First isolated in Tanzania in 1952 [18], CHIKV is transmitted by mosquito vectors of the *Aedes (Stegomyia)* subgenus and has caused a number of outbreaks in Africa and Asia during the last 50 years [19]. It is believed that the original natural history of viral transmission relies on virus maintenance in a yellow fever-like zoonotic sylvatic cycle involving non-peridomestic mosquitoes and nonhuman primates, as previously described in Africa. However, explosive urban outbreaks were associated with a dengue-like direct “human-mosquito-human” transmission cycle implicating *A. aegypti* or more recently *A. albopictus* mosquitoes [20]. Particularly large CHIKV outbreaks have occurred in Indian Ocean islands, in India, and in Southeast Asia since 2005 [6]. All these epidemics originated from east Africa and were associated with the East-Central-South African genotype [21]. Significantly, these recent epidemics are also associated with viral transmission by *A. albopictus*, although this was previously considered a secondary vector, and convergent adaptation to this mosquito has been observed in different geographical regions [22]–[23]. Approximately 40% of the population of Reunion Island was infected (around 300,000 people) [24], including a relatively small proportion of severe disease in adults and newborns, as well as some vertical transmission [25]–[26]. Worryingly, the increasingly widespread distribution of *A. albopictus* may result in CHIKV epidemics in more temperate regions. Indeed, in 2007, a CHIKV outbreak occurred in Italy, and the detection of two autochthonous cases in France in 2010 confirmed fears of the possible expansion of this important viral disease [27]–[28], particularly as there is currently no commercialized antiviral or vaccine for this virus.

Codon re-encoding may represent an important tool for the design of effective vaccines for CHIKV and other arboviruses. However, for such a strategy to succeed it is essential to determine how the virus might respond to this profound change in selection pressure, particularly given its reliance on both mosquitoes and mammals for transmission. Key questions include: to what extent will codon re-encoding reduce viral fitness, how rapidly will CHIKV recover fitness following re-encoding, and will this fitness recovery involve direct reversion at synonymous sites? To address these questions, we studied the *in cellulo* replicative fitness of codon re-encoded CHIKV and the *in cellulo* evolution of re-encoded viruses by combinations of either alternate or continuous passage of each virus in primate or insect cells.

## Results

Using a large scale random re-encoding method which randomly attributed nucleotide codons based on their corresponding amino acid sequence (e.g. the amino acid valine was randomly replaced by GTT, GTC, GTA or GTG; see materials and methods for more details), we designed, synthesized, and incorporated into a WT infectious clone (IC; derived from the LR2006 CHIKV strain) three re-encoded cassettes of around 1.4 Kb, located in the non-structural proteins nsP1, nsP4, and in a region overlapping the structural proteins E2 and E1. A total of 264, 298 and 320 synonymous mutations were present in these re-encoded regions, respectively. Six re-encoded viruses were studied using combinations of these re-encoded regions: Φnsp1, Φnsp4 and Φenv with one re-encoded region; Φnsp1 Φnsp4, and Φnsp4 Φenv with two re-encoded regions and Φnsp1 Φnsp4 Φenv with three re-encoded regions (**Figure S1 in Text S1**). Their genetic characteristics, such as G+C%, codon usage bias, frequencies of rare codons and of the dinucleotides CpG/UpA, remained comparable with WT virus and 132 other CHIKVs from GenBank (**Table S1 in Text S1**).

### Replicative fitness of the re-encoded viruses

The WT virus and the six re-encoded viruses were derived following transfection of the corresponding infectious DNA clones into Vero cells. Viruses were then passaged once in Vero cells and their replicative fitness was studied. All the viruses produced a cytopathic effect (CPE) which was delayed proportionally with the degree of re-encoding (from 2 days for the WT virus to 5–6 days for the Φnsp1 Φnsp4 Φenv virus).

#### (i) Single cycle replication kinetics and plaque morphology

**Figure 1:**
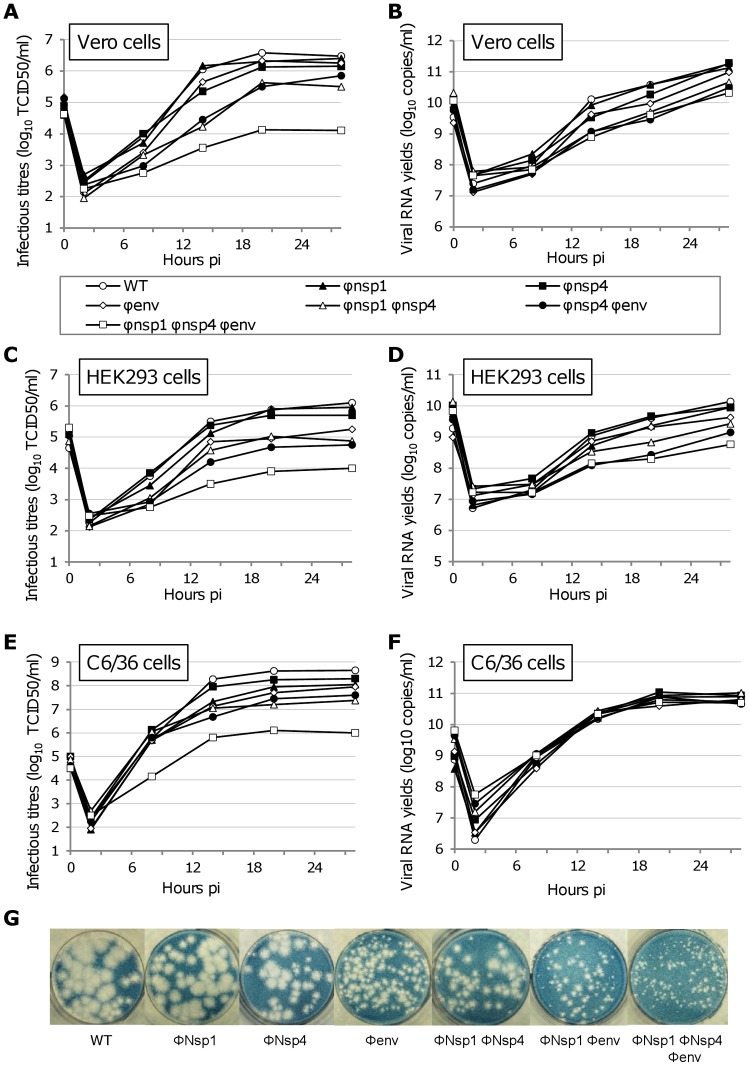
Single cycle replication kinetics and plaque morphologies using WT and re-encoded viruses. To study replicative fitness of the re-encoded viruses, all viruses (including the WT) were used to perform single cycle replication kinetics in Vero (**A–B**) HEK293 (**C–D**) and C6/36 cells (**E–F**). An estimated MOI of 5 was used to infect confluent cells. Cells were washed 30 minutes after the infection and 1 ml of cell supernatant was sampled at several time pi. Clarified (centrifugation) cell supernatants were used to measure (i) the infectious titres using a TCID50 assay (**A, C, E**) and (ii) the amount of viral RNA using a real time RT-PCR assay (**B, D, F**). The plaque morphology of these viruses was also analysed comparatively in Vero cells (**G**).

Using primate (Vero and HEK293) and mosquito cell lines (C6/36), replication kinetics were investigated using a high estimated multiplicity of infection (MOI = 5) (**Figure 1**) during the first 28 hours post-infection (pi). Quantitative analyses were performed at 8 and 14 hours pi (**Table S2 in Text S1**), i.e. a period shorter than two complete replication cycles on the basis of 8 hours per replication cycle [17].

When analysis of virus replication in primate or mosquito cells was based on infectious titres in cell supernatants (as measured using TCID50 method), an inverse linear relationship was observed between infectious titres at 14 hours pi and the number of synonymous mutations in re-encoded regions (**Figure S2 in Text S1**). Detailed results are reported in **Table S2 in Text S1** and summarised below: For the Φnsp1 Φnsp4 Φenv virus, infectious titres were significantly lower than those of the wild type virus. The difference reached 1 to 1.5 and 2 to 2.5 log_10_ TCID50/ml at 8 and 14 hours pi, respectively. For viruses with two regions re-encoded (Φnsp1 Φnsp4 and Φnsp1 Φenv), significant infectious titre reductions compared with WT virus (0.5 to1 log_10_ TCID50/ml) were observed for all constructs propagated in C6/36 cells and for the Φnsp1 Φnsp4 virus in Vero cells at 8 hours pi. All titres were significantly lower at 14 hours pi (1–1.5 log_10_ TCID50/ml). For viruses with one region re-encoded (Φnsp1, Φnsp4 and Φenv), significant infectious titre reductions, compared with WT, were observed for the Φenv virus in HEK293 cells (∼0.5–1 log_10_ TCID50/ml at 8 and 14 hours pi) and the Φnsp1 and Φenv viruses in C6/36 cells (∼0.75–1.25 log_10_ TCID50/ml at 14 hours pi). However, compared with WT virus, there were no significant reductions of infectious titres in Vero cells at any time.

When replication was assessed on viral RNA yields (as measured using quantitative real time RT-PCR and cell supernatants), strikingly different results were observed in mosquito and primate cells (**Figure 1** and **Figure S2 in Text S1**). In C6/36 mosquito cells, viral RNA yields remained identical regardless of the degree of re-encoding. In primate cells, an inverse linear relationship between RNA yield at 14 hours pi and the number of synonymous mutations in re-encoded region(s) was observed.

**Figure 2:**
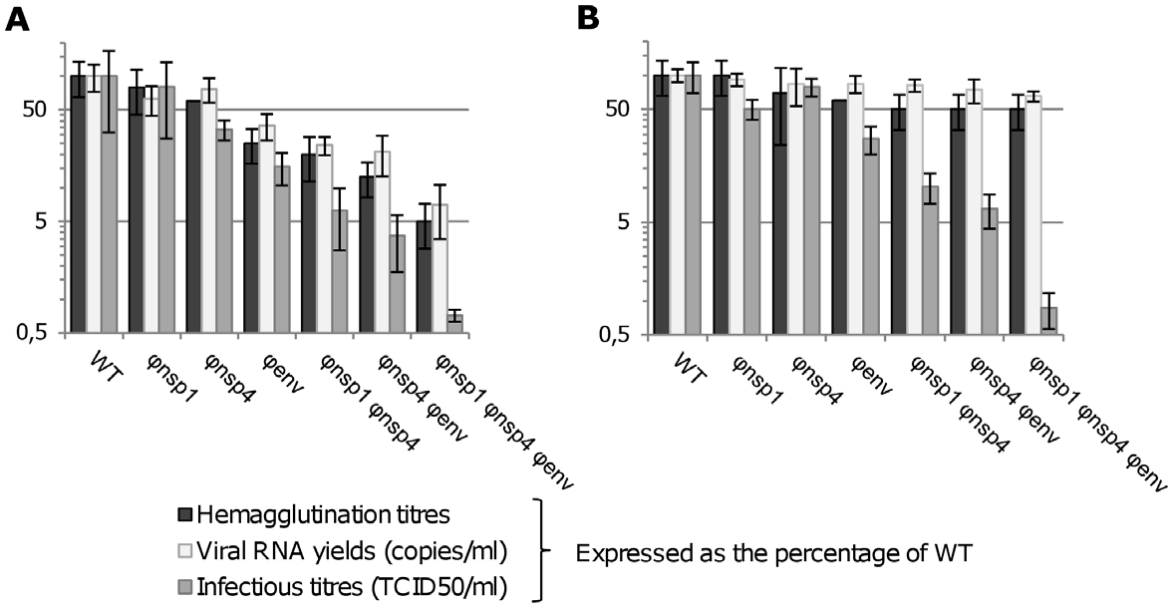
Comparison of the results from haemagglutination, real time RT-PCR and TCID50 assays. To assess the relationship between viral RNA yields and the number of viral particles in the cell supernatants, the results of quantitative RT-PCR, TCID50 and haemagglutination assay were compared. An estimated MOI of 5 was used to infect confluent Vero (**A**) or C6/36 (**B**) cells. Cells were washed after the infection and cell supernatants were sampled at 16 hours pi. Clarified (centrifugation) cell supernatants were used to perform haemagglutination, real time RT-PCR and TCID50 assays. Results are expressed in the graphs as percentage of WT and are the mean/standard deviation of three independent experiments.

To assess the relationship between viral RNA yields and the number of viral particles (i.e. infectious and non infectious particles) in the clarified supernatants, results of the quantitative RT-PCR assay, the TCID50 assay and a haemagglutination assay were compared. The latter provided an estimate of the relative quantity of CHIKV haemagglutinin. This, in turn, reflects the number of viral particles [29]–[30], and provides a direct comparison between WT and re-encoded viruses which encode identical proteins. At 16 hours pi, using an estimated MOI of 5, viral RNA yields and haemagglutinin titres from both Vero and C6/36 cell supernatants showed parallel production kinetics. On the other hand, TCID50 titres progressively decreased with increasing levels of re-encoding in both primate and mosquito cells (**Figure 2**). This result implies that the real-time RT-PCR assay reflects the number of viral particles and hence that the re-encoding does not appear to impact significantly on the incorporation of viral RNA into viral particles.

In C6/36 cells, as the degree of codon re-encoding increased, the number of viral particles remained relatively stable. However, infectious titre values gradually decreased up to 2.5 log_10_ TCID50/ml (**Figure 1** **and Table S2 in Text S1**). This indicates that re-encoding was associated with a significant modification of the infectivity of viral particles (i.e. the ratio of the number of infectious particles [calculated here using a TCID50 assay] over the number of viral particles [calculated here using a quantitative PCR assay]). When compared with the WT virus, we observed a 500 fold decrease of infectivity for the most re-encoded (Φnsp1 Φnsp4 Φenv) virus, 30–50 fold for viruses with two regions re-encoded and 5–25 times for viruses with one region re-encoded (**Table S2 in Text S1**).

Primate cells also showed a decrease of viral infectivity as the degree of re-encoding increased. However, this level of decrease was more limited, reaching a 10–20 fold reduction for the most re-encoded virus (**Table S2 in Text S1**). By contrast with the C6/36 cells, the production of viral particles was significantly decreased at 14 hours pi for viruses with 3 regions (∼1 log_10_ RNA copies/ml) and 2 regions re-encoded (0.5–1 log_10_ RNA copies/ml) (**Figure 1** **and Table S2 in Text S1**). In addition, each virus with one region re-encoded showed a significant reduction at 14 hours pi in one primate cell type at least (approximately 0.5 log_10_ RNA copies/ml). Therefore, whilst the major modification observed in C6/36 cells was a reduction of virus infectivity yields, in primate cells there was also a reduction of the number of viral particles.

The plaque morphology in Vero cells confirmed the results of the single cycle replication kinetics: globally, as the degree of re-encoding increased, there was a corresponding decrease in plaque size (**Figure 1**). Interestingly, the Φenv virus plaque size most closely resembled that of viruses with two re-encoded regions rather than Φnsp1 or Φnsp4 virus.

#### (ii) Replication kinetics with low MOI viral infection or infectious DNA clones

**Figure 3:**
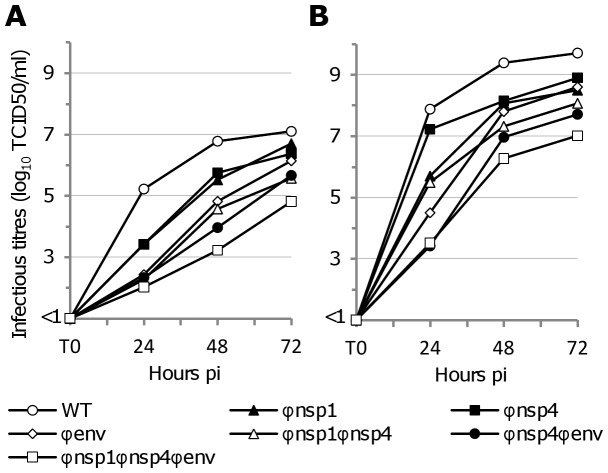
Replication kinetics using low MOI with WT and re-encoded viruses. An estimated MOI of 0.01 was used to infect confluent Vero (**A**) or C6/36 (**B**) cells. Cells were washed and cell supernatant was sampled at several times pi. Clarified (centrifugation) cell supernatants were used to measure the infectious titres using a TCID50 assay.

Defective interfering (DI) particles are known to impact on viral replication cycles [31]. To assess their potential role in the differences observed in our single cycle replication studies, we analysed the kinetics of virus production using a low estimated MOI (0.01) in Vero and C6/36 cells (**Figure 3**). The infectious titre values decreased with increasing degrees of re-encoding, regardless of the observation time pi, indicating that DI particles did not play a major role in the effect observed using high estimated MOI. Globally, the degree of reduction of virus infectivity in comparison with the WT virus was higher than that observed in the single cycle replication kinetics (approximately 3–4 log_10_ TCID50/ml for the most re-encoded virus 24 hours pi). This presumably reflects the fact that these analyses were performed following several viral replication cycles, thus amplifying the difference observed in the single-cycle experiments reported earlier (**Figure 3**).

**Figure 4:**
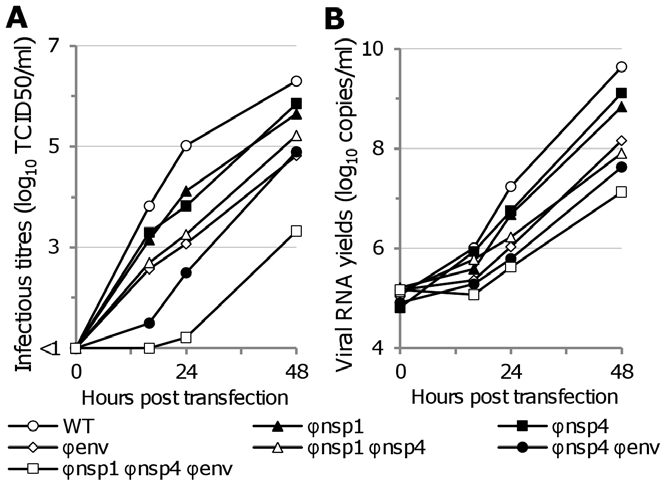
Replication kinetics using infectious DNA clones with WT and re-encoded viruses. Equal quantities of ICs were used to transfect culture flasks of HEK293 cells. Cells were washed and cell supernatant was sampled at several time pi. Clarified (centrifugation) cell supernatants were used to measure (i) the infectious titres using a TCID50 assay (**A**) and (ii) the amount of viral RNA using a real time RT-PCR assay (**B**).

Using a different method we confirmed that the observed effect could not be due to higher numbers of DI particles in cell supernatants derived from re-encoded viruses: ICs were directly used to transfect HEK293 cells. Infectious titres and number of viral particles were then measured in cell supernatants (**Figure 4**). The infectious titre values decreased with the degree of re-encoding, regardless of the time pi. At 16 hours pi, the infectious titre of the most re-encoded virus was undetectable whilst values of the WT virus were around 4 log_10_ TCID50/ml. As observed in single cycle replication kinetics, the number of viral particles using RNA yields as the indicator of particle numbers decreased less than the infectious titres (**Figure 4**).

In comparison with the single cycle replication experiments, when compared with WT virus, both methods identified a significant reduction of infectious titres for each of the three viruses with one region re-encoded (Student's *t* test, all *p* value<0.01 with low estimated MOI 48 hours pi and DNA clones 24 hours pi). As with plaque morphology in Vero cells, we observed that the replicative fitness of the Φenv virus more closely resembled viruses with two regions re-encoded than that of Φnsp1 or Φnsp4 virus (**Figure 3** **–** **4**).

#### (iii) Competition experiments

**Figure 5:**
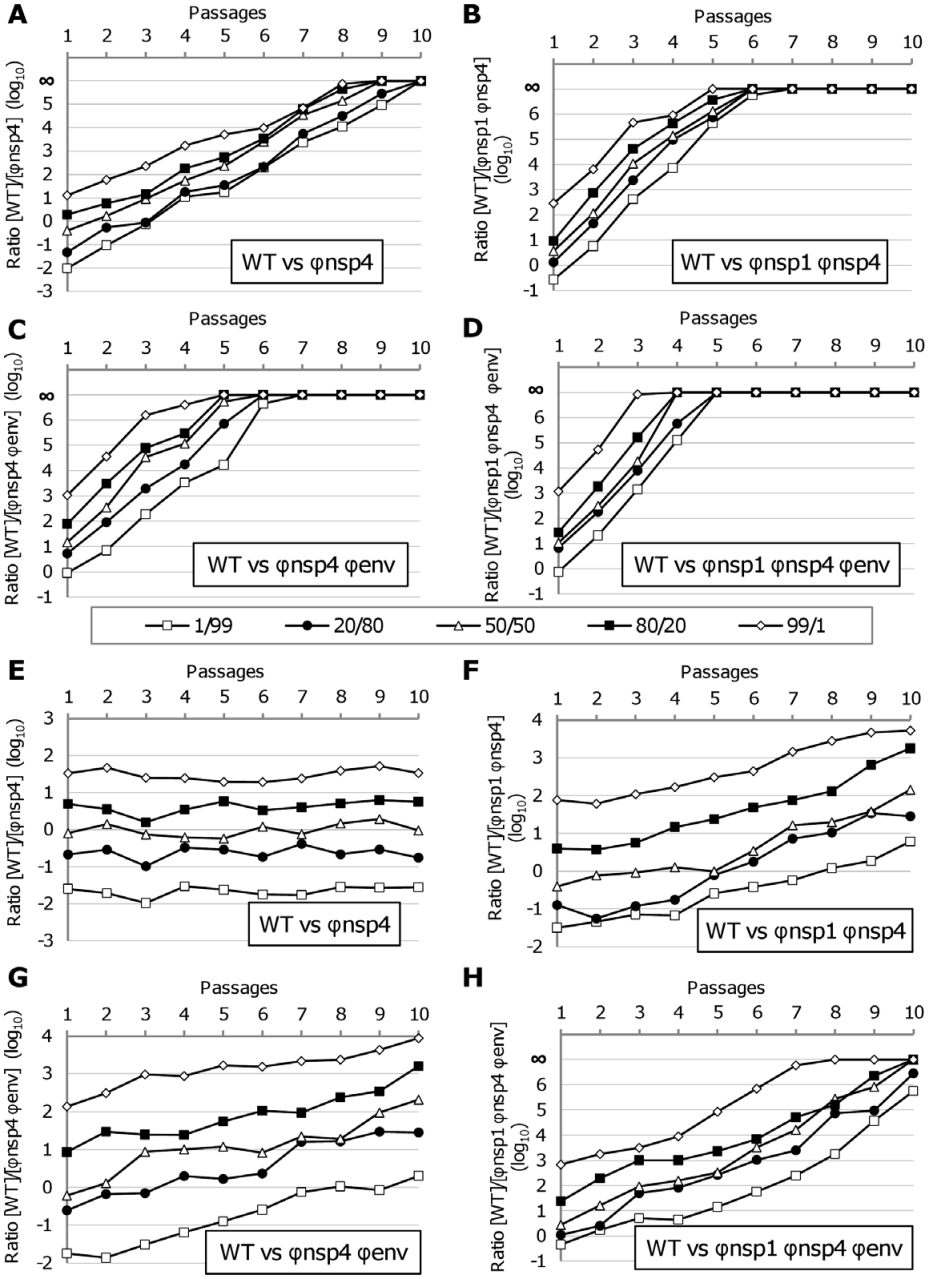
Competition experiments. Using two real time RT-PCR assays, specific for the WT virus or the viruses with the nsP4 region re-encoded (Φnsp4, Φnsp1 Φnsp4, Φnsp4 Φenv and Φnsp1 Φnsp4 Φenv viruses), competition experiments were performed in Vero (**A–D**) and C6/36 (**E–H**) cells. Five initial PFU ratios (WT/re-encoded virus: 1/99, 20/80, 50/50, 80/20, 99/1) were used. This enabled us to quantify the proportion of each virus in the viral population (expressed as log_10_ WT/re-encoded ratio). In addition, cell supernatants were passaged 10 times to enable long term follow-up of each viral population.

Using two real time RT-PCR assays, specific for the WT virus or the viruses with the nsP4 region re-encoded (Φnsp4, Φnsp1 Φnsp4, Φnsp4 Φenv and Φnsp1 Φnsp4 Φenv), competition experiments were performed in Vero and C6/36 cells. Five initial PFU ratios (WT/re-encoded virus: 1/99, 20/80, 50/50, 80/20, 99/1) were used. As described in the Methods section, this procedure provided quantitative estimates of the proportion of each viral genome in the viral population (expressed as log_10_ WT/re-encoded ratio in **Figure 5**). In addition, cell supernatants were passaged 10 times to enable long term follow-up of each viral population (viruses completed an estimated 60 replication cycles).

In Vero cells (**Figures 5A–D**), WT virus was proportionally more fit than re-encoded viruses depending on the degree of re-encoding. Re-encoded viruses disappeared completely after 8–10, 5–7 and only 3–5 passages for the Φnsp4 virus, the viruses with two re-encoded regions and the most re-encoded virus respectively. Two extreme initial PFU ratios were tested in Vero cells with the Φnsp4 virus (0.1/99.9 and 0.01/99.99) and viral populations followed a similar pattern, with WT virus rapidly supplanting the re-encoded virus (Data not shown). In C6/36 cells (**Figures 5E–H**), WT virus was also proportionally more fit than re-encoded viruses depending on the degree of re-encoding but this process occurred more slowly than in Vero cells: WT/Φnsp4 ratios remained stable during 10 passages, whereas ratios for the viruses with two re-encoded regions increased by ∼2–2.5 log_10_ and the Φnsp1 Φnsp4 Φenv virus was rapidly supplanted by the WT virus.

#### (iv) Synthesis of viral proteins and intracellular viral RNA

**Figure 6:**
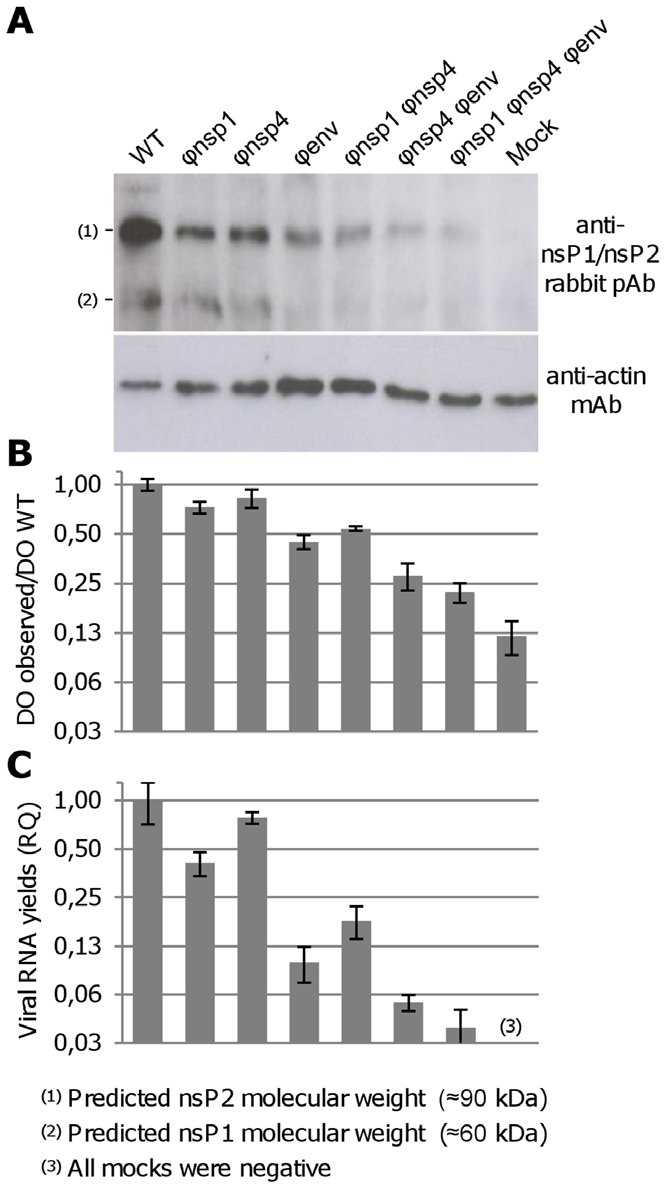
Synthesis of viral proteins and intracellular viral RNA. To study, at the intracellular level, how our codon re-encoding method modified viral RNA and protein synthesis, 12-well-plates of HEK293 cells were infected with virus at an estimated MOI of 5. Cells were washed after the infection and intracellular RNA and proteins were extracted at 8 hours pi simultaneously or individually (cf. materials and methods). Viral protein levels were measured using two methods: a western blot analysis using anti-nsP1/nsP2 rabbit pAb (**A**) and an ELISA (**B**) using a CHIKV-specific immune human serum (recognizing especially the structural proteins [32]–[35]). In panel **B**, results are expressed as percentage of WT and are the mean/standard deviation of three independent experiments. Viral RNA yields were quantified by real time comparative RT-PCR using actin mRNA as a normalizer and cells infected by the WT virus as a calibrator (**C**); Results are the mean relative quantity (RQ) from three independent experiments and errors bars are RQ max and RQ min values generated by the Stratagene Mx3005P software at 99% confidence level; Results are the mean and standard deviation from three independent experiments.

All the experiments described above incorporated clarified infectious cell supernatant medium to study the replicative fitness of re-encoded viruses. However, we also studied these parameters at the intracellular level by assessing how the codon re-encoding method modified intracellular viral RNA and protein yields. Because the presence of stable levels of viral RNA and HA in the supernatant of the C6/36 cells indicated that RNA and protein synthesis was unaffected by re-encoding, only human cells were studied in this analysis. HEK293 cells were infected with a high estimated MOI (5.0), and intracellular RNA and proteins were extracted at 8 hours pi. Viral protein levels were measured by western blot analysis using anti-nsP1/nsP2 rabbit pAb and ELISA using a CHIKV-specific immune human serum (recognizing especially the structural proteins [32]–[35]; **Protocol S1**). Viral RNA yields were measured using a real time RT-PCR assay (**Figure 6**).

Both methods revealed a quantitative decrease in the level of structural and non-structural viral protein production with increasing degrees of codon re-encoding (around five times for the most re-encoded virus using ELISA). Viral RNA yields also decreased with increasing degrees of codon re-encoding (around 25 times for the most re-encoded virus) (**Figure 6**). These results confirmed the supernatant medium analysis (**Figure 1**) and implied that in human cells, re-encoding compromised the replication complex resulting in decreased levels of viral RNA and viral protein in cells and supernatant medium.

### Experimental passages of the WT and two re-encoded viruses

After their recovery by transfection, two re-encoded viruses (Φnsp4 and Φnsp1 Φnsp4 Φenv) and the WT virus were passaged using three different protocols: 50 serial passages in non-human primate (Vero) or mosquito (C6/36) cells, and 50 alternate passages in Vero and C6/36 cells (*i.e.*, 25 double passages (Vero/C6/36 cells)). At the time of each passage, the estimated MOI was bottlenecked at approximately 0.1 to minimize the generation of defective interfering particles without generating a major population bottleneck. Each passage was terminated after 48 hours. It is therefore estimated that each virus completed ∼300 replication cycles after fifty passages on the basis of 8 hours per replication cycle [17].

#### Fitness of passaged CHIKV in non-human primate and insect cells

**Figure 7:**
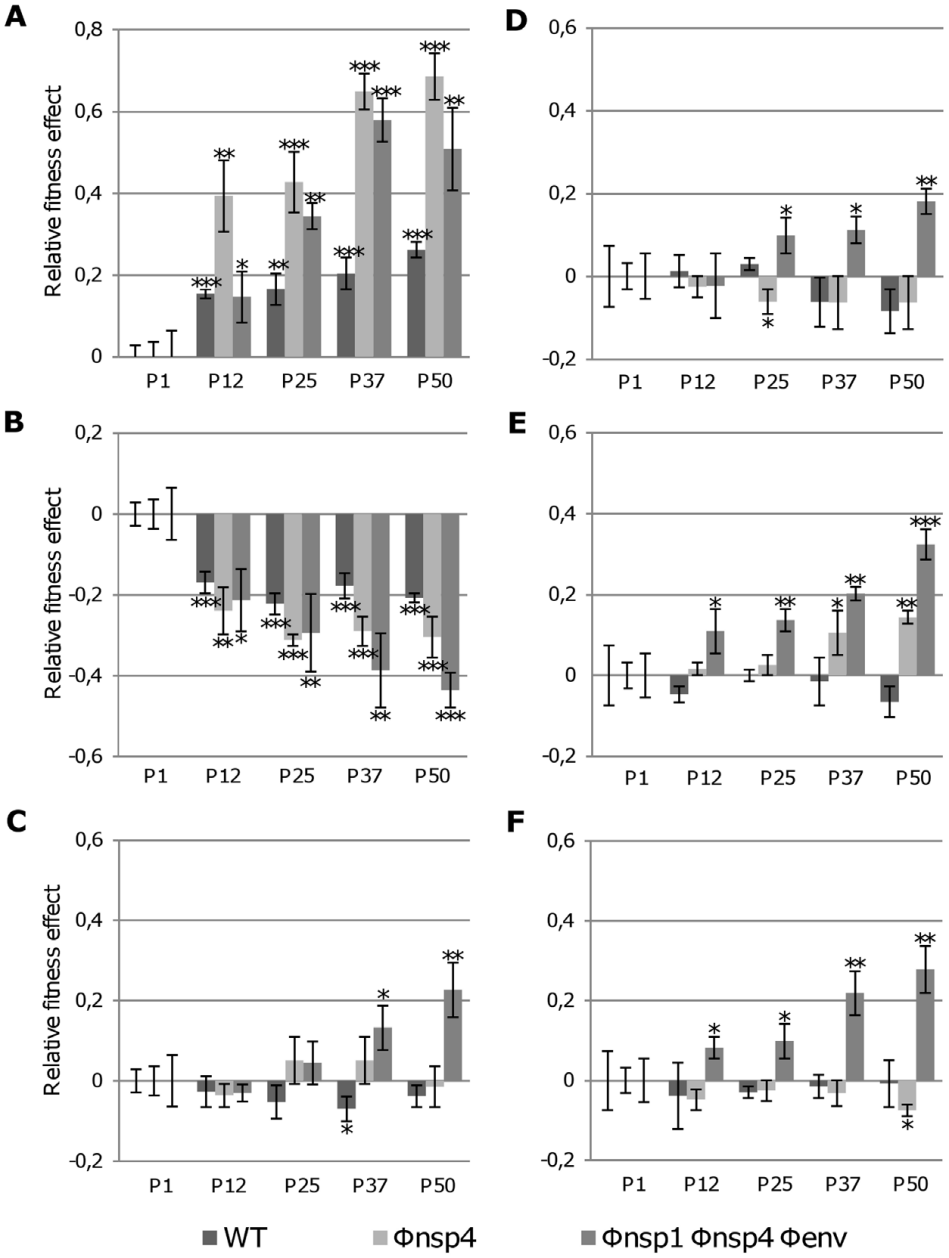
Fitness modifications observed during the passage experiments. The replicative fitness of each virus effect is represented here using relative fitness effect values. These values represent: [(the viral growth rate of a passaged virus at 24 hours pi, based on TCID50 values)/(the viral growth rate of the corresponding virus at the first passage)]−1 (See **Materials and Methods**). The relative fitness effect was calculated using replication kinetics experiments in Vero cells (**A–C**) and C6/36 cells (**D–F**). Panels **A** and **D** represent viruses serially passaged in Vero cells, **B** and **E** viruses serially passaged in C6/36, and **C** and **F** viruses alternately passaged. Significant values (comparison made with the first passage of the same virus in the same cells, Student's *t* test) are indicated by * (0.01<*p*<0.05), ** (0.001<*p*<0.01 and *** (*p*<0.001).

To study the evolution of replicative fitness during passages in response to codon re-encoding, we examined the replicative kinetics of each passaged virus at the 1^st^, 12^th^, 25^th^, 37^th^ and 50^th^ passage in Vero and C6/36 cells (**Figure S3, S4, S5 in Text S1**). We first measured the viral growth rate at 24 hours pi, based on TCID50 values. The corresponding relative fitness effect values are detailed in **Figure 7**. We then performed a global analysis of TCID50 values at 24, 48 and 72 hours pi by performing two-way repeated-measures ANOVA and tukey's HDS post-hoc comparisons (**Table S3 in Text S1**), which provided very similar results.

First, we showed that the fitness trajectories of WT virus changed with the passaging methods. This phenomenon has been widely reported in the arbovirus literature [36]–[40]. Replicative fitness in Vero cells significantly increased following serial passage in Vero cells, whereas it decreased following serial passage in C6/36 cells and slightly decreased in the case of alternate passages (**Figure 7**). Replicative fitness in C6/36 cells remained globally unchanged following serial passage in C6/36 cells, in Vero cells or both. Φnsp4 behaviour appeared to be similar to that of the WT virus, with the exception of replicative fitness in C6/36 cells which significantly increased after serial passage in these cells.

In the case of Φnsp1 Φnsp4 Φenv, the evolution of the replicative fitness was strikingly different: it increased in all cases except in Vero cells after serial passage in C6/36 where a significant decrease was observed (**Figure 7**). Thus, in most cases, the replicative fitness of the re-encoded viruses increased significantly during serial passage whereas that of the WT virus remained unchanged. In addition, after 25 serial passages in Vero cells, both re-encoded viruses showed an increase of their replicative fitness in Vero cells which is significantly higher than that observed for the WT virus (Student's *t* test, all *p* value <0.01).

Despite these specific replicative fitness gains, the infectious titres of the Φnsp1 Φnsp4 Φenv virus in each cell type did not approach those obtained with the first passage of the WT virus: the smallest difference observed at 24, 48 and 72 h pi was 2.6, 1.9 and 0.7 log_10_ TCID50 in Vero cells, and 1.7, 0.9 and 0.5 log_10_ TCID50 in C6/36 cell, respectively (**Figure S3, S4, S5 in Text S1**).

#### Genome sequence analysis of passaged CHIKV

To understand which genomic modifications accompanied these changes in replicative fitness, the complete genome consensus nucleotide sequences of Φnsp4 and Φnsp1 Φnsp4 Φenv were established at passage 50. Intra-population viral genetic diversity was assessed in three coding regions (nsP2/nsP3, E2 and E1): approximately 20 clones were sequenced for each PCR product studied (mean: 19.2+/−4.2; range from 14 to 44). Because each virus passage was not repeated, and in view of the globally low number of mutations detected, no statistical analysis of the data was performed in this section.

**Figure 8:**
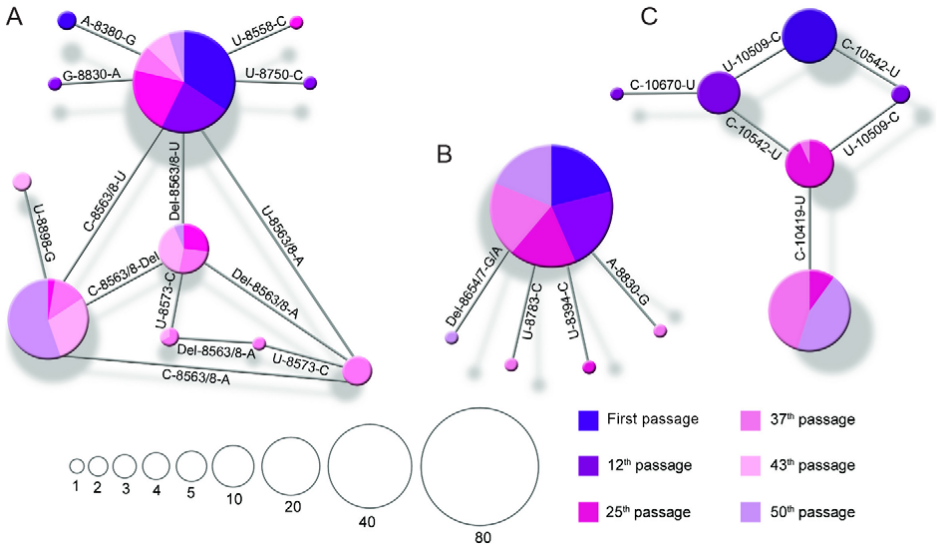
Intra-population genetic diversity of CHIKV. Minimum spanning trees were used to depict the intra-population genetic diversity of CHIKV. Each panel represents all the clones sequenced in one region of one virus passage from one method: (**A**) E3/E2 region of the Φnsp4 virus passaged in C6/36 cells, (**B**) E3/E2 region of the Φnsp1 Φnsp4 Φenv virus alternatively passaged, and (**C**) E1 region of the Φnsp1 Φnsp4 Φenv virus serially passaged in Vero cells. Each circle represents one variant and its size corresponds to the number of clones with the same nt sequence. The original sequence is represented by the biggest circle except in panel (c) where it is the circle at the top. Mutation positions are indicated in each branch. For the point mutations, the nt present in each viral population is shown. For the 6 nt deletion found in the viruses serially passaged in C6/36 cells at nt positions 8556/61 (panel **A**) which was considered as a unique event, (i) the word ‘Del’ indicates that it is present in the nearest viral population, (ii) no modification compared to the original sequence are represented by U, (iii) C and A indicate the presence of the 8566u>c and 8566u>a mutations, respectively.

**Figure 9:**
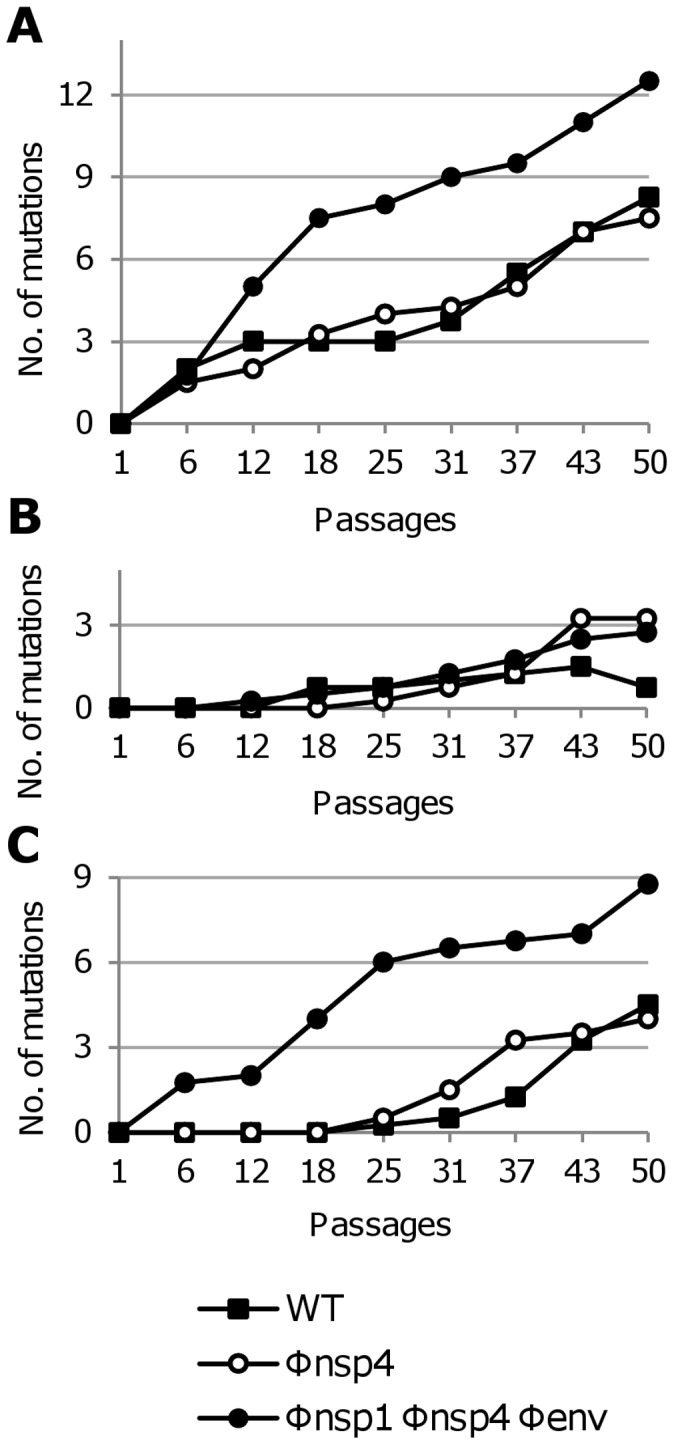
Mutation emergence kinetic curves. For (**A**) the virus serially passaged in Vero cells, (**B**) serially passaged in C6/36 cells, and (**C**) alternatively passaged, curves represent the number of mutations detected in complete genome consensus sequences during the passages. These graphics take into account the fixation rate of each mutation corresponding to the presence of mixed viral populations detected when double peaks were observed on the sequencing chromatograms (**Table S4 in Text S1**). Numbers are as follows: 1 for a fixed mutation, 0.75 for <, 0.5 for ≈, 0.25 for > and zero when the mutation was not yet detected.

In our experimental model of evolution, selection pressure applied to the re-encoded viruses arose from both codon re-encoding and adaptation to culture conditions. All the viruses, re-encoded or not, followed similar evolutionary pathways according to passaging method. First, we noted an association between the number of observed mutations in complete genome consensus sequences and the passage method: highest after serial passage in Vero cells (range 8 to 16), lowest after serial passage in C6/36 cells (range 3 to 6), and intermediate after alternate passage (range 6 to 10) (**Table 1**). Very few mutations were detected during serial passage in C6/36 cells, all of these were non-synonymous and most (85%) were located in the structural protein coding region (**Table 1** and **Table S4 in Text S1**). Moreover, among the few mutations observed after the serial passages in C6/36 cells, only 1/13 became fixed in the population (based on complete genome consensus sequences), while among those observed after serial passage in Vero cells and alternate passage,19/34 and 9/23 mutations, respectively, were fixed (**Table 1**). This observation was confirmed by analysis of intra-population genetic diversity: (i) fluctuations in the size of variant sub-populations were observed with viruses serially passaged in C6/36, and (ii) mutations observed after serial passage in Vero cells and alternate passage tended to be fixed on a more regular and rapid basis (**Figure 8** and **Figure S6 in Text S1**). Furthermore, from the 6^th^ passage onwards, viruses serially passaged in Vero cells exhibited a continuous appearance of mutations, whereas those passaged in C6/36 cells exhibited a very low mutation frequency, and a delayed emergence of mutations was observed for alternately passaged viruses; in the latter case mutations were first detected at the 25^th^ passage for both the WT and Φnsp4 viruses (**Figure 9**). Our experiments also revealed frequent convergent evolution; a total of 15 and 24 convergent mutations were observed using complete genome consensus and clonal sequences, respectively (**Table 2**). Approximately 50% of these convergent mutations were shared between re-encoded viruses and the WT virus. Notably, all those convergent mutations found in complete genome consensus sequences occurred in viruses passaged using the same method or between serially and alternatively passaged viruses (**Table 2**), denoting adaptation to cell culture conditions.

**Table 1:** Characteristics of the mutations detected in the complete genome consensus sequences of the passaged viruses.

Virus	Passage method	No. of mutations	Point mutations				No. of deletions	Reversions			No. of fixed mutations
			total	5′UTR	S	NS		No.	Freq.	Rate	
WT	Vero	10	10	0	2	8	0	-	-	-	5
	C6/36	3	2	0	0	2	1	-	-	-	0
	Alternate	6	6	0	1	5	0	-	-	-	3
Φnsp4	Vero	8	8	0	4	4	0	1	12.5%	0.34%	6
	C6/36	6	4	0	0	4	2	0	0%	0%	0
	Alternate	7	7	0	1	6	0	0	0%	0%	0
Reenc^3^	Vero	16	16	2	7	7	0	2	12.5%	0.23%	8
	C6/36	4	4	0	0	4	0	0	0%	0%	1
	Alternate	10	10	2	2	6	0	0	0%	0%	6

WT, Φnsp4 and Reenc^3^ represent the WT, Φnsp4 and Φnsp1 Φnsp4 Φenv viruses passaged 50 times using the method described in the column entitled ‘Passage method’.

5′UTR designates mutations in the 5′ untranslated region, S synonymous mutations and NS non-synonymous mutations.

**Table 2:** Shared mutations detected in complete genome consensus sequences and in viral sub-populations via cloning.

Nt position	Region	Nt change	AA change	Viruses sharing the mutation when based on:	
				Consensus sequences	Sub-populations
22	5′UTR	A→G	-	Reenc^3^-VERO, Reenc^3^-ALT	
66	5′UTR	A→G	-	Reenc^3^-VERO, Reenc^3^-ALT	
822	nsP1	C→U	P→L	Reenc^3^-VERO, Reenc^3^-ALT	
3218	nsP2	U→G	S→A	Φnsp4-ALT, Reenc^3^-ALT	
4167	nsP3	G→U/A	G→V/D	Φnsp4-ALT	WT-ALT, Reenc^3^-C6/36
6330	nsP4	U→C	V→A	Φnsp4-VERO, Reenc^3^-ALT	
6426	nsP4	C→U	A→V	WT-VERO, WT-ALT, Reenc^3^-ALT	
8563-8	E2	Del-GUCUAU	Del-VY	WT-C6/36, Φnsp4-C6/36	Reenc^3^-C6/36
8566	E2	U→C/A	Y→H/N	WT-C6/36, Φnsp4-C6/36	Reenc^3^-C6/36
8731	E2	U→C/A	W→R	WT-VERO, WT-ALT	WT-C6/36, Φnsp4-ALT
8831	E2	U→C	M→T	Reenc^3^-C6/36	WT-C6/36
8836	E2	C→U	H→>Y	WT-VERO	WT-C6/36
10,509	E1	C→U	-	Reenc^3^-VERO	Reenc^3^-C6/36
10,542	E1	C→U	-	Reenc^3^-VERO	Reenc^3^-ALT
10,670	E1	U→C	V→A	WT-C6/36, WT-ALT, Φnsp4-C6/36, Φnsp4-ALT, Reenc^3^-C6/36, Reenc^3^-ALT	Reenc^3^-VERO
3641	nsP2	G→A/C	V→L	-	Φnsp4-C6/36, Reenc^3^-ALT
3897	nsP2	G→A	C→>Y	-	WT-C6/36, WT-ALT
8394	E3	C→U	-	-	WT-C6/36, Reenc^3^-ALT
8558	E2	U→C	F→S	-	WT-C6/36, Φnsp4-C6/36
8573	E2	C→U	A→V	-	Φnsp4-C6/36, Φnsp4-ALT, Reenc^3^-C6/36
8750	E2	U→C/A	M→K/T	-	WT-C6/36, Φnsp4-C6/36
8830	E2	A→G	M→V	-	ΦNSP4-C6/36, Reenc^3^-ALT
8898	E2	G→U	R→S	-	ΦNSP4-VERO, ΦNSP4-C6/36
8783	E2	C→U	A→V	-	ΦNSP4-ALT, Reenc^3^-ALT

WT, Φnsp4 and Reenc^3^ represent the passaged WT, Φnsp4 and Φnsp1 Φnsp4 Φenv viruses. Viruses are represented by their name followed by ‘VERO’ for serial passages in Vero, ‘C6/36’ for serial passages in C6/36, and ‘ALT’ for alternate passages. All the mutations detected in complete genome consensus sequences during the passages were used (**Table S4 in Text S1**) as well as those used for the analysis of intra-population genetic diversity (**Figure 8** **and Figure S6 in Text S1**).

5′UTR designates the 5′ untranslated region.

Of note, a six nucleotide deletion at positions 8562/72 which was found in the three viruses serially passaged in C6/36 (twice in the complete genome consensus sequences and once using cloning methods), and there was a rapid increase in frequency of a 9 nt deletion at positions 4139/47 in Φnsp4 (from 0% to 60% of the overall population in only 13 passages). This indicates that acquisition of deletion mutations constituted an important mechanism for adaptation to C6/36 cells (**Table S4 in Text S1**). In addition, complex evolutionary pathways were observed during passages in C6/36 cells: some sub-populations acquired mutations – such as 8573c>u, 8831u>c and 8563_8568del – which then disappeared partially or completely during subsequent passages (**Figure 8** and **Figure S6 in Text S1**).

The re-encoded viruses, particularly the Φnsp1 Φnsp4 Φenv virus, were also associated with specific evolutionary patterns. Serial passages in Vero cells resulted in a higher number of mutations in the Φnsp1 Φnsp4 Φenv virus (16 versus 10 and 8 for the WT and Φnsp4 viruses, respectively) and an increased proportion of synonymous mutations in both re-encoded viruses (50% versus 20% for the WT virus) (**Table 1**). During alternate passages for the Φnsp1 Φnsp4 Φenv virus, there was also an early appearance and fixation of mutations (**Figure 9**), whilst at the 50^th^ passage, all alternately passaged viruses displayed a similar total number of mutations (**Table 1**). These specific evolutionary patterns indicate that despite the adaptation to culture conditions shared by the WT and re-encoded viruses, a proportion of the mutations that emerged during serial passage in Vero cells appeared in response to codon re-encoding. The fact that the Φnsp1 Φnsp4 Φenv virus serially passaged in Vero cells showed significantly increased replicative fitness in both Vero cells and C6/36 cells, reinforces this conclusion.

To detect the mutations specific to codon re-encoding, we first hypothesized that adaptation to culture conditions and response to codon re-re-encoding may have occurred sequentially. However, the fact that some mutations associated with codon re-encoding appeared very early (*e.g.*, 22a>g, 66a<g, 10509c>u and 10542 c>u, see below) suggested that both phenomena occurred concurrently.

When those convergent mutations shared only between re-encoded viruses were analysed (**Table 2**), the early emergence of two identical mutations located in the 5′untranslated region (5′UTR) (22a>g and 66 a>g) was identified for the Φnsp1 Φnsp4 Φenv viruses serially passaged either in Vero cells or alternately in both cells. This suggested that these mutations induced major fitness changes in response to codon re-encoding (both mutations were already detected at the third passage for both viruses). In addition, two convergent non-synonymous mutations were identified within the re-encoded nsP1 (822c>u) and nsP4 (6330u>c) regions, suggesting that non-synonymous mutations could arise in response to codon re-encoding (**Table 3**) in the case of viruses passaged in Vero cells or alternately in both cells. No convergent mutation was identified in C6/36 passaged viruses.

**Table 3:** Mutations that emerged in response to codon re-encoding and detected using complete genome consensus sequences.

Nucleotide position	Region	Nucleotide change	AA change	Reversion	In a re-encoding region	Virus(es) harboring the mutation
22	5′UTR	A→G				Reenc^3^-VERO, Reenc^3^-ALT
66	5′UTR	A→G				Reenc^3^-VERO, Reenc^3^-ALT
742	Nsp1	A→U	-		+	Reenc^3^-ALT
822	Nsp1	C→U	P→L		+	Reenc^3^-VERO, Reenc^3^-ALT
6330	Nsp4	U→C	V→A		+	Φnsp4-VERO, Reenc^3^-ALT
6670	Nsp4	C→A	N→K		+	Φnsp4-C6/36
6717	Nsp4	A→C	E→A		+	Reenc^3^-VERO
6761	Nsp4	A→G	T→A		+	Reenc^3^-VERO
6971	Nsp4	U→C	-	+	+	Φnsp4-VERO
9591	E2	U→G	-		+	Reenc^3^-ALT
9855	6K	A→G	-		+	Reenc^3^-VERO
10419	E1	U→C	-	+	+	Reenc^3^-VERO
10509	E1	C→U	-	+	+	Reenc^3^-VERO
10542	E1	C→U	-		+	Reenc^3^-VERO
10896	E1	C→U	-		+	Reenc^3^-VERO

Φnsp4 and Reenc^3^ represent the passaged Φnsp4 and Φnsp1 Φnsp4 Φenv viruses. Viruses are represented by their name followed by ‘VERO’ for serial passages in Vero, ‘C6/36’ for serial passages in C6/36, and ‘ALT’ for alternate passages.

Eleven mutations were identified specifically in the re-encoded regions of genetically modified viruses (**Table 3**). These included (i) 3 reversions: 2 for Φnsp1 Φnsp4 Φenv virus and 1 for Φnsp4 virus passaged in Vero cells; (ii) 5 additional synonymous mutations observed for Φnsp1 Φnsp4 Φenv virus passaged in Vero cells or alternately in both cells; (iii) 3 non-synonymous mutations, of which 2 appeared in Φnsp1 Φnsp4 Φenv virus passaged in Vero cells and 1 in Φnsp4 virus passaged in C6/36 cells.

In summary, despite the substantial specific replicative fitness gains observed for the re-encoded viruses, there was a marked absence of reversion mutations for viruses passaged in C6/36 cells and alternately in vertebrate/invertebrate cells, and only a low reversion rate (<0.4%) in Vero cells (**Table 1**). Rather than re-introducing their original codons through synonymous back mutations, the re-encoded viruses largely acquired non-synonymous mutations (61%) or novel synonymous mutations (22%).

In C6/36 cells, only one mutation candidate in response to codon re-encoding (6670c>a) was identified for the Φnsp4 virus despite the significant replicative fitness changes observed (**Table 3**). By contrast, in Vero cells 12 mutation candidates could be identified, including a cluster of six synonymous mutations in the Φenv re-encoded region. This specific response to codon re-encoding probably reflects the greater decrease in replicative fitness observed for the Φenv virus in comparison to other viruses with one region re-encoded (see above).

**Figure 10:**
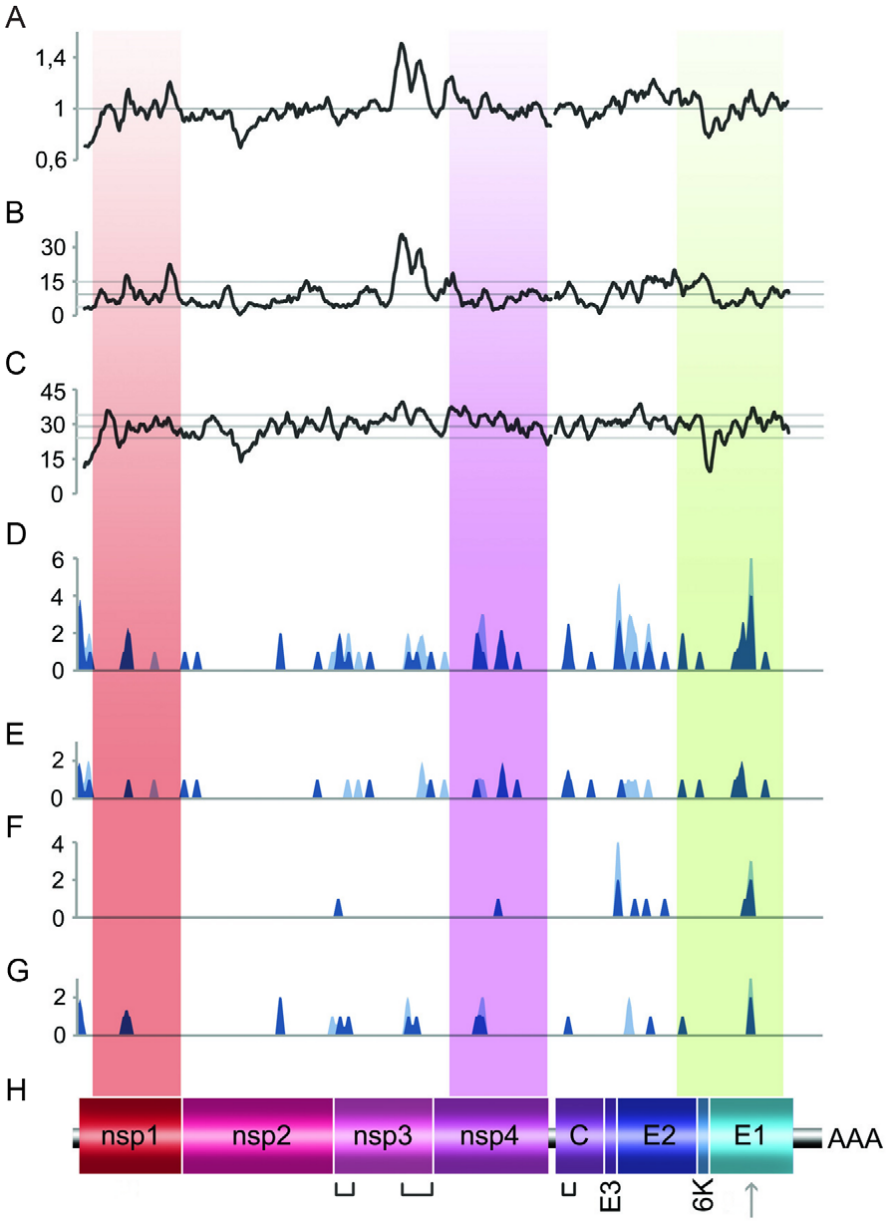
Mutation distributions. Patterns of mutation distribution in 132 CHIKV extracted from GenBank (**A–C**) and of the passaged viruses (**D–G**), all of them aligned with the CHIKV complete genome sequence (**H**). Using the alignment of the CHIKVs from GenBank, the variability at (**A**) 1^st^+2^nd^+3^rd^ (**B**) 1^st^+2^nd^ codon and (**C**) 3^rd^ codon positions were determined and represented using 150, 100 and 50 nt sliding windows, respectively. (**A**): 1 represents the mean value from all sites; (**B–C**): values represent the number of variable nt positions in the sliding window interval; mean +/− standard deviations were also represented). Using complete genome consensus sequences from (**D**) all passaged viruses, (**E**) viruses serially passaged in Vero cells, (**F**) viruses passaged in C6/36 cells or (**G**) viruses alternatively passaged, the sum of mutations detected at each nt position (deletions were considered as one unique mutation) was determined and represented using 100 nt sliding window. All the mutations are represented in light blue and mutations from re-encoded viruses were superimposed in dark blue. Shaded regions indicate the three re-encoded regions. From left to right, brackets indicate the X-domain and the hypervariable C-terminal region of the nsP3 and the capsid binding RNA region. The grey arrow indicates the E1-V226A mutation.

The distribution of mutations along the genome observed for re-encoded viruses partly matched that of the WT virus (a probable consequence of the adaptation to the same culture conditions), but also that of genetic variability observed in CHIKV genomes retrieved from GenBank (**Figure 10**). Passaged viruses exhibited the same highly variable regions and some of these mutational hot spots (*e.g.*, the hypervariable C-terminal region of the nsP3 [17] and the E2 region) were also highly variable amongst GenBank CHIKV sequences. These regions were associated with high levels of amino acid diversity in passaged viruses (**Table S4 in Text S1**) and other CHIKVs (**Figure 10**). Conversely, regions of low variability amongst the GenBank CHIKV genomes were associated with a reduction in synonymous variability, particularly within nsP1, nsP2 and E1, and were also invariable in all passaged viruses (except the nsP1 region). Nevertheless, some mutational hot spots found in all passaged viruses were relatively invariable amongst GenBank CHIKV genomes, notably the X-domain of the nsP3 [41], and specific regions in nsP1, E2/E1 and the capsid binding RNA region [17] (**Figure 10** and **Table S4 in Text S1**).

Finally, the mutation at position 10670 (E1-V226A) was observed in all viruses passaged in C6/36 cells and alternately in Vero and C6/36 cells but was fixed only once (during serial passages in C6/36 of Φnsp1 Φnsp4 Φenv virus). This confirms that the A226V mutation, a strong inducer of adaptation to *A. albopictus* (**Figure 10**), does not provide a significant fitness advantage for replication in the C6/36 *A. albopictus* cell culture system [19], [22].

## Discussion

We have evaluated the effect on replicative fitness and cytopathogenicity of large-scale re-encoding of CHIKV, a re-emerging Old World pathogenic arbovirus. The generation of attenuated viruses by large-scale re-encoding represents an exciting and potentially important route to vaccine development, and also to understanding the basis of the evolution of viral pathogenicity. Site-directed re-encoding, associated with no modification of amino acid sequences, alleviates the likelihood of novel phenotypic properties, allows us to modulate fitness by altering the length of the codon replacement interval, but additionally provides benefits to the generic development of live attenuated vaccines, including reduced costs and single dose induction of long-term immunity [42].

A key result was the observation that our random re-encoding method decreased the replicative fitness of CHIKV in both primate and arthropod cells. The diminution of CHIKV replicative fitness correlated directly with the degree of re-encoding. As reported in previous studies with unrelated viruses, we found that during one replicative cycle in mosquito cells, codon re-encoding profoundly reduced the infectious titre of released virus whilst the number of viral particles remained stable [12], [14]. This implies that the maturation process (i.e. the formation of ribonucleoproteins and their insertion into plasma membranes that contain HA) could be at fault when viruses are re-encoded. In contrast, in primate cells, this decline in infectivity of the viral particles was associated with the reduced generation of viral RNA and proteins probably due to a compromised replication complex. Because they can be identified at different stages of the CHIKV replication cycle, these results imply that the observed decrease in replicative fitness is probably the consequence of several independent re-encoding induced events. However, it is important to note that (i) alphaviruses produce very different kinds of infection in mosquito and primate cells (i.e. persistent infection in mosquito cells and cytolytic infection in vertebrate cells), and (ii) there are differences in host cell response and innate immunity between mosquito and primate cells, and which could also explain these observed differences in the different cell lines [17], [43]–[45].

There is mounting evidence that synonymous mutations in viral genomes may have major fitness effects and not only in the small number of cis-acting elements described previously [46]. In the current study, six re-encoded viruses were produced of which the most re-encoded virus modified in three regions that encode different proteins (together, 882 synonymous mutations were introduced spanning 4,212 nt). In support of previous studies which demonstrated that re-encoded poliovirus and influenza A viruses are attenuated [10]–[14], our observations of a reduction in replicative fitness strongly suggest that a proportion of synonymous mutations are not neutral in RNA viruses. Indeed, it is likely that some synonymous mutations were positively selected during the passaging process, reinforcing the idea that synonymous sites are central to viral fitness. In conclusion, it is likely that synonymous mutations can be either neutral, beneficial or deleterious as is the case for non-synonymous mutations.

Evolutionary patterns at synonymous sites could be shaped by genome-wide mutational processes, such as G+C%, codon usage bias and dinucleotide frequency [8], [47]–[49]. These global constraints, which theoretically produce a subset of viable genomes, were assessed by previous studies of codon re-encoding in poliovirus, influenza A virus and bacterial virus T7 which applied specific modification of codon usage bias, codon pair bias or CpG/UpA frequencies [10]–[15]. Using a large-scale random re-encoding method, which only slightly modified these global properties, we still observed replicative fitness reductions in both primate and arthropod cells. Our results suggest that local constraints may also provide significant selection pressure on synonymous sites in RNA viruses, for example by disrupting RNA secondary structures. Since numerous functional secondary structures are present in coding regions of RNA viruses, and hence include synonymous sites (with notable examples in poliovirus [50], tick-borne encephalitis virus [51], alphaviruses [17], [52] and HIV-1 [53]), it is likely that similar structures are common in CHIKV. Recently, it was demonstrated that a similar re-encoding strategy applied to the noncapsid regions of the poliovirus resulted in the identification of two novel functional RNA elements [54]. The concept of large-scale random re-encoding, as described here, is also supported by the report of the negative impact of random single synonymous mutations (which did not modify the genetic characteristics of the genome) on viral replicative fitness [46].

Finally, our results and those of previous studies of re-encoded viruses [10]–[14] suggest that the reduction of viral replicative fitness is driven by a variety of factors. First, the nature of the virus studied is an important parameter: we found that introducing up to 882 random synonymous mutations clearly affected the replicative fitness of the CHIKV, whilst two previous studies demonstrated that comparable random re-encoding methods applied to the capsid precursor (P1) region of the poliovirus did not significantly affect replicative fitness (934 [14] and 153 [11] synonymous substitutions were introduced, respectively). The location of the re-encoded region constitutes the second factor of importance: re-encoding in the E2/E1 region resulted in a greater loss of fitness than in other genomic regions. The analysis of complete wild type CHIKV genomes revealed naturally low levels of synonymous diversity in this re-encoded region (**Figure 10**) indicating that this region is subject to specific local evolutionary constraints which in part explain the significant impact of re-encoding in this region. The re-encoding method applied is obviously an additional important parameter: previous studies with polioviruses showed that reduction of replicative fitness was strongly dependent on the method used to re-encode the genome [11]–[14].

The average impact of one mutation is clearly likely to be less important in random re-encoding than in specific approaches [11]–[14]. This suggests that random large-scale re-encoding could be advantageous in several aspects when designing future vaccine candidates, namely: *(i)* reversion to wild-type should be intrinsically more difficult, given the high number of mutations produced; *(ii)* since in our experiments the reduction of replicative fitness decreased with the degree of re-encoding, the method opens the door to finely tuning fitness reduction through modulation of the length of re-encoded regions and the number of synonymous mutations introduced; *(iii)* the use of a combination of several re-encoded regions located throughout the viral genome may prevent complete phenotypic reversion due to recombination between WT and re-encoded viruses: large scale sequence modification may render recombination intrinsically more difficult, and in the case of recombination, the part of the genome representing the re-encoded strain would likely still carry some mutations associated with fitness reduction.

Taken together, these observations suggest that, following large-scale random re-encoding, recovery of the original replicative fitness should require a large number of reversion mutations. Consequently these re-encoded viruses should be very stable [12], [14]. To test this hypothesis and to study the constraints that shape CHIKV codon usage, we passaged the wild type and two re-encoded CHIKVs *in cellulo*. It is commonly stated that arboviruses are subject to strong evolutionary trade-offs, such that mutations that are favoured in one host are deleterious in another, and that this imposes constraints on viral evolution [16], [37], [39]. This implies that a strain that has been adapted to a mammalian cell line should have a reduced replicative fitness in mosquito cells, and *vice versa*. Consequently, we could have initiated our experiments *(i)* with a strain previously adapted to a given cell line, with the disadvantage of introducing an obvious bias in other cell lines (*i.e.*, follow a strategy inspired by Bull *et al.* who used a pre-adapted bacterial virus T7 [12], [15]), or *(ii)* with a clinical strain, *i.e.* isolated from previous alternate passages in mosquitoes and humans, with the disadvantage of possibly observing a simultaneous response to codon re-encoding and adaptation to culture conditions (as performed by Burns *et al.* who used a non-adapted poliovirus [12], [15]). We chose the latter, based on the observation that no criteria existed for defining the adaptation period in the case of arboviruses. This choice was retrospectively justified by the observation that the follow-up of replicative fitness and molecular evolution could not distinguish the criteria necessary for differentiating both phenomena.

A key observation of our study was that few reversion mutations occurred, despite specific replicative fitness enhancements in response to codon re-encoding. This suggests that the effectiveness of large-scale re-encoding methods results from the accumulation of slightly deleterious mutations that push the virus into a fitness valley, and that there are multiple opportunities through diverse mutational pathways, sometimes in genomic regions other than those we re-encoded, in which these viruses can partially restore their fitness. For example, amongst the mutation candidates emerging in response to codon re-encoding, two were in the 5′ UTR. That these mutations were fixed so rapidly is strongly suggestive of their selectively beneficial effect [55]. Moreover, it was notable that although we only made modifications to synonymous sites, some of the mutations observed were non-synonymous. Therefore, the evolution of these viruses in response to codon re-encoding was largely compensatory in nature and very few mutations were the result of reversion. Moreover, even with the specific fitness enhancements in response to codon re-encoding observed, the most re-encoded virus failed to reach fitness levels (infectious titres) equivalent to those observed at the first passage of the WT virus. Interestingly, these fitness improvements were not always accompanied by greater numbers of mutations or specific molecular modifications. For example, during alternate passage, the most re-encoded virus succeeded in increasing its fitness without accumulating more mutations than other viruses (*i.e.*, WT and the less re-encoded viruses).

During serial passage of the re-encoded viruses, we observed that the response to codon re-encoding and adaptation to culture conditions occurred simultaneously. However, the high levels of observed convergent evolution between the WT virus and the re-encoded viruses indicates that selection arising from codon re-encoding was likely weaker than that for adaptation to culture conditions, and/or that the beneficial mutations to restore the cost of re-encoding were less likely to arise. Therefore, this indirect insight into the difficulty of reversing the effects of re-encoding further highlights the stability of these re-encoded viruses.

Our experiments also confirm that mutations acquired in one host can be deleterious in a different host type (serial passages in primate cells increased viral replicative fitness in primate cells, whilst serial passages in mosquito cells decreased viral fitness in primate cells) and, with the exception of the most re-encoded virus, that alternate passages seriously (i) limit replicative fitness enhancement, and (ii) delay the appearance of the mutations. It is noteworthy that replicative fitness in mosquito cells remained globally unchanged following serial passage in mosquito cells, in Vero cells or in both. Moreover, as described previously with dengue virus [38], replication in mosquito cells appears to act as a brake on viral evolution; in our case, very few mutations were detected after 50 passages and only one was fixed, suggesting that the majority of the emerging mutants have a deleterious effect on viral fitness. In addition, we observed weaker selection pressure in these cells during competition experiments. The fact that the C6/36 cell line was selected more than 30 years ago for its capacity to replicate CHIKVs and dengue viruses [56] could explain this weaker selection pressure observed. Conversely, the rapid adaptation of CHIKV to *A. albopictus* was accompanied by multiple appearances of the E1-A226V mutation [19], [22]–[23] and the appearance of amino-acid deletions in the nsP3 and E2 genes.

In conclusion, this study demonstrates that random codon re-encoding significantly decreases the replicative fitness of CHIKV. Although all these results are important and encouraging, they cannot be easily extended to RNA viruses producing chronic infections. Thus, studies in animal models are obviously needed to evaluate the potential of these new generation attenuation methods for producing vaccine candidates. However, this approach could assist in the development of future RNA virus vaccines, including those for arboviruses. Introducing a large number of slightly deleterious synonymous mutations reduced the replicative fitness of CHIKV by orders of magnitude in both primate and arthropod cells. This strategy resulted in limited reversion and recovery of fitness after intensive serial subculture of the viruses, and is likely to reduce the risk of complete phenotypic reversion if recombination with wild type virus occurs. Our results encourage us that such modified viruses would find it difficult to return to their natural arboviral cycle in the real world. Furthermore, the decrease of the replicative fitness correlated with the extent of re-encoding, an observation that may be advantageous in the development of future strategies to modulate viral attenuation.

## Materials and Methods

Cell culture protocols for virus stock production and virus titration (TCID50, plaque and haemagglutination assays) are detailed in **Protocol S1**.

### In s*ilico* re-encoding method

Three regions of the CHIKV genome were re-encoded using a computer program that randomly attributed nucleotide codons based on their corresponding amino acid sequence: for example, the amino acid valine was randomly replaced by GTT, GTC, GTA or GTG. To minimize the influence of rare codons in primate cell lines, the number and the position of such rare codons in primate genomes [57] (i.e. CGU, CGC, CGA, CGG, UCG, CCG, GCG, ACG) were not modified. In addition, unique restriction sites were conserved by correcting synonymous mutations at some sites. The location of the re-encoded cassettes, first based on the availability of unique restriction sites was adjusted to avoid overlap with known RNA secondary structures [17], [52]. Finally, three cassettes of 1302, 1410 and 1500 bases and located in the nsP1, nsP4 and E2/E1 regions, respectively, were designed using this method (**Text S2**).

### Construction of CHIKV infectious clones (ICs)

We modified a previously described IC of the LR2006 strain of CHIKV [58] (GenBank accession EU224268) and all the re-encoded regions were synthesized (GenScript) and then inserted into ICs as described in **Protocol S1**. Using a combination of re-encoded regions, six re-encoded ICs were generated: Φnsp1, Φnsp4 and Φenv with one re-encoded region; Φnsp1 Φnsp4, and Φnsp4 Φenv with two re-encoded regions and Φnsp1 Φnsp4 Φenv with three re-encoded regions (**Figure S1 in Text S1**).

### Real time RT-PCR assays

A fragment of 179 nt located in the nsP2 region (nucleotide position 2631 to 2809) was used to detect the genomic RNA (plus strand) of all the CHIKVs (universal assay), re-encoded or not. Another fragment of 168 nt located in the nsP4 region (nucleotide position 6804 to 6971) was used to analyze cell supernatants from competition experiments: two sets of primers and probes allowed us to specifically detect either the viruses re-encoded in the nsP4 region or the viruses without modification in the same region. Primer and probe sequences and the real time PCR protocol are detailed in **Table S6 in Text S1** and **Protocol S1**, respectively.

### Replication kinetics

The replicative fitness of each virus was determined using the results of replication kinetics studies, performed in triplicate in Vero, HEK293 or C6/36 cells. For comparison of the seven viruses from the seven ICs (the WT virus and the 6 re-encoded viruses), one experiment was performed with all the viruses. Virus stock (see **Protocol S1**) or ICs were used to infect or transfect cells respectively. For the evaluation of replicative fitness of the passaged viruses, we performed one experiment for each virus (WT, Φnsp4 and Φnsp1 Φnsp4 Φenv viruses) with the first passage in Vero and the 12^th^, 25^th^, 37^th^ and 50^th^ passages for each passage regimen (13 supernatants tested in triplicate). For the single cycle replication kinetics (**Figure 1**), an estimated MOI of 5 was used to infect a 75 cm^2^ culture flask of confluent Vero, C6/36 or HEK293 cells. Cells were washed twice (HBSS) 30 minutes after the infection and 20 ml of medium was added. 1 ml of cell supernatant was sampled just before the washes and at 2, 8, 14, 20 and 28 hours pi. For the replication kinetics with low estimated MOI (**Figure 3**) and the evaluation of the replicative fitness of the passaged viruses (**Figure S3, S3, S5 in Text S1**), an estimated MOI of 0.01 was used to infect a 25 cm^2^ culture flask of confluent Vero or C6/36 cells. Cells were washed twice (HBSS) 2 hours after infection and 8 ml of medium was added. 1 ml of cell supernatant was sampled after the washes (T0) and at 24, 48 and 72 hours pi. For the replication kinetics using infectious DNA clones (**Figure 4**), a 75 cm^2^ culture flask of subconfluent HEK293 cells was transfected with the ICs using Lipofectamine 2000 (Invitrogen) according to the manufacturer's instructions. Cells were washed twice (HBSS) 4 hours after the transfection and 20 ml of medium was added. 1 ml of cell supernatant was sampled after the washes (T0) and at 16, 24 and 48 hours pi.

All the sampled cell supernatants were clarified by centrifugation, aliquoted and stored at −80°C. They were then analysed using a TCID50 assay and a real time RT-PCR assay (not performed systematically, see figure legends). Nucleic acids were extracted from clarified cell supernatants using the EZ1 Virus Mini Kit v2 on the EZ1 Biorobot (both from Qiagen).

### Virus competition experiments

WT virus was grown in competition with one of four re-encoded viruses (Φnsp4, Φnsp1 Φnsp4, Φnsp1 Φenv or Φnsp1 Φnsp4 Φenv) using five different PFU ratios (WT/re-encoded virus 1/99, 20/80, 50/50, 80/20, 99/1). A global estimated MOI of 0.5 was used for the first inoculation. For each experiment, a 25 cm^2^ flask culture of confluent cells was infected for 2 hours, washed (HBSS) and then incubated for 48 h after the addition of 7 ml of medium. Viruses from each experiment were then passaged nine times as follows: a 25 cm^2^ flask culture of confluent cells was infected for 2 hours with the purified culture supernatant (centrifugation), washed (HBSS) and then incubated for 48 h after the addition of 7 ml of medium. At each passage, the estimated MOI was bottlenecked at approximately 0.5. After each infection, nucleic acids were extracted from the clarified culture supernatant using the EZ1 Virus Mini Kit v2 on the EZ1 Biorobot (both from Qiagen). Using two specific real time RT-PCR assays targeting the ΦnsP4 region (see above), the amount of each virus was assessed and the ratio of the two values (WT/re-encoded) was calculated.

### Quantification of intracellular RNA and viral proteins

A global estimated MOI of 5 was used to infect confluent 12 well-plates of HEK293 cells with virus stock (see **Protocol S1**). Cells were washed once (HBSS) 30 minutes after the infection and 2 ml of media was added. At 8 hours pi, the absence of cytopathic effect was checked, culture supernatants were discarded, and cells were washed once (HBSS). All experiments were performed in triplicate. For Western blot analysis and intracellular viral RNA quantification, total RNA and protein isolation was performed using the same well with the Nucleospin RNA/protein kit according to the manufacturer's instructions (Macherey-Nagel). Protein extracts were resolved on 10% polyacrylamide gels containing SDS and transferred to PVDF membrane. Anti-Nsp1/2 rabbit pAb (see **Protocol S1**), anti-actin C-2 mAb (Santa Cruz Biotechnology) and the corresponding HRP-conjugated secondary antibody were used. Protein bands were revealed using Immobilon (Millipore) followed by exposure of blot to radiographic film. Real time RT-PCR assay (see above) was performed to assess viral intracellular RNA (mRNA actin was used as a normalizer to account for differences in cells number and/or quality of extracted RNA as described previously [59]). For the quantification of viral proteins by ELISA, cells were mechanically harvested using a cell scraper, resuspended in 800 µL of PBS, vortexed and disrupted by sonication (30 seconds at 20 KHz, Misonix Sonicator XL). Pre-treated CHIKV-specific immune human serum was used to detect viral proteins. ELISA protocol is detailed in **Protocol S1**.

### Experimental passage of viruses *in cellulo*

The WT and two re-encoded viruses (Φnsp4 and Φnsp1 Φnsp4 Φenv) were passaged 50 times following three regimens: serial passages in Vero or C6/36 cells and alternate passages between Vero and C6/36. For each passage, a 25 cm^2^ culture flask of confluent cells was infected for 2 hours with the diluted clarified cell supernatant, washed (HBSS) and incubated for 48 hours after the addition of 7 ml of medium. Cell supernatant was then harvested, clarified by centrifugation, aliquoted and stored at −80°C. For each passage, the estimated MOI was bottlenecked at approximately 0.1. To avoid contamination, virus passages were performed in three phases: serial passages of WT and Φnsp4 viruses, alternate passages of the same viruses and passages of the Φnsp1 Φnsp4 Φenv virus. All the viruses passaged at the same time were manipulated sequentially and in different laminar flow cabinets.

### Consensus sequencing

Whole genome nucleotide sequences (excluding the first 18 nucleotides of the 5′UTR and the 22 nucleotides upstream of the polyA tail) were determined for all the 50^th^ passage viruses (nine viruses in total). The timing of emergence of each mutation found in the 50^th^ passage was then determined by sequencing with appropriate primer pairs for the 6^th^, 12^th^, 18^th^, 25^th^, 31^st^, 37^th^ and 43^rd^ passages. To avoid contamination by PCR products and plasmids, we utilized a molecular biology laboratory that is specifically designed for clinical diagnosis using molecular techniques, and which includes separate laboratories dedicated to perform nucleic acid extraction, PCR/RT mix, RNA/cDNA manipulations and PCR products/plasmids manipulations. In addition, each step from extraction to sequencing or cloning of PCR products (see below) was performed in separate experiments for each passaged virus. Nucleic acids were extracted from the purified culture supernatant using the EZ1 Virus Mini Kit v2 on the EZ1 Biorobot (both from Qiagen). A set of specific primer pairs (**Table S5 in Text S1**) was used to generate amplicons with the Access RT-PCR System (Promega) according to the manufacturer's instructions. PCR products were then purified and sequenced using forward and reverse primers with the BigDye Terminator v3.1 Cycle Sequencing Kit on an ABI Prism 31310X Genetic Analyser sequencer (both from Applied Biosystems). Analysis of sequencing chromatogram and combination of sequences was performed using the Sequencher 4.9 software (Gene Codes Corporation).

### Intra-population genetic diversity

Three genomic regions were chosen for analysis: one of 619 nt overlapping the nsP2 and nsP3 regions (positions 3601 to 4220), one of 619 nt overlapping the E3 and E2 regions (positions 8351 to 8970), and one of 740 nt located in the E1 region (positions 10140 to 10880). The 1^st^, 12^th^, 25^th^, 37^th^, 43^rd^ and 50^th^ passages of each passaged virus were chosen to assess the intra-population genetic diversity in the E3/E2 region, while the nsP2/nsP3 and E1 regions were analyzed in the passaged Φnsp1 Φnsp4 Φenv viruses (using the same passage numbers as above). In addition, the 12^th^, 25^th^, 37^th^, 43^rd^ and 50^th^ passages of the Φnsp4 virus serially passaged in C6/36 were also analyzed in the nsP2/nsP3 region to study the emergence of the 4139_4147del mutation. The AccuScript *PfuUltra* II RT-PCR Kit (Agilent) was used according to the manufacturer's instructions to generate amplicons from extracted nucleic acids (see above). PCR products were cloned after purification into the StrataClone PCR Cloning Vector and transformed into competent cells (StrataClone PCR Cloning Kit; Agilent). A plasmid extraction was performed from bacterial colonies with the correct insert which had been previously cultured and plasmid DNA was automatically sequenced with a T7 primer (GATC Biotech). Finally, approximately 20 clones were sequenced for each PCR product analyzed (mean: 19.2+/−4.2; range from 14 to 44), resulting in a total of 315, 933 and 227 sequenced clones for the nsP2/nsP3, E3/E2 and E1 regions, respectively.

### Evolutionary analysis

Complete viral genome consensus sequences were manually constructed and aligned. Base ambiguity symbols were used to represent all the mixed viral populations when double peaks were observed on the sequencing chromatograms (as represented ≈, > and < in **Table S4 in Text S1**). The 132 CHIKV complete genome nucleotide sequences already available in GenBank (**Text S2**), along with the outgroup O'Nyong-Nyong virus (ONNV) strain SG650 (GenBank accession AF079456) sequence, were extracted from GenBank/NCBI. The two ORFs of these viruses were manually extracted and concatenated using the Bioedit v7.0.9 program [60] and aligned with ClustalW [61] according to the amino acid sequence. Ambiguously aligned regions were removed manually. This alignment was used to estimate the variability at each nt position using the Mega5 software [62]–[63].

All the sequences obtained to assess the intra-population genetic diversity were edited using the Sequencher 4.9 software (Gene Codes Corporation). Using the Mega5 software [62], all the sequences from the same region were aligned and each mutation which was found only once in the comparative alignment (i.e. singletons) was removed to ensure that no mutations introduced during the RT-PCR were included in the analysis: 22, 49 and 15 singletons were removed from the nsP2/nsP3, E3/E2 and E1 alignments, respectively. Minimum spanning trees of clonal CHIKV data were constructed using the TCS1.21 software [64] with a 99% connection limit. For each virus and primer pair, we used the alignment of all the sequences (see above) after removing singletons. Each deletion (6 or 9 nt) was considered to be a unique event irrespective of length. When a mutation occurred in a deleted region (detected in another clone), it was assigned the position of the corresponding deletion.

### Estimation of the relative fitness effect and statistical analysis

To study the evolution of replicative fitness during the serial subculture of viruses we performed replicative kinetics studies at the 1^st^, 12^th^, 25^th^, 37^th^ and 50^th^ passage of each virus in Vero and C6/36 cells (**Figure S3, S3, S5 in Text S1**). Data from these kinetic studies were analyzed using two different methods.

We first estimated the relative fitness effect values (**Figure 7**): we measured the viral growth rate at 24 hours pi, based on TCID50 values, to calculate the relative fitness effect as described previously [65]. This measure has to be performed during the viral exponential growth phase. Because most of the infectious titre values obtained with the Φnsp1 Φnsp4 Φenv virus at 24 hours pi were close to the detection threshold of our TCID50 assay, we chose the 24 hours pi values for all the viruses, even though some of the values of the WT and Φnsp4 viruses reached a plateau before 24 hours (**Figure S3, S4, S5 in Text S1**). Titres at time *t_0_* (all the values were under the detection threshold of our TCID50 assay and then considered as zero) and at *t_1_* (24 hours pi) were used to calculate the growth rate (*r*) as the increase in log-titre per 24 hours. Relative fitness (*W*) was defined as the growth rate ratio and the relative fitness effect as *s = W−1*. The relative fitness for each experiment *i* was calculated as 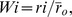 where 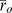 is the average of three determinations for the first passage of the virus in the same cells. We then performed a global analysis of TCID50 values at 24, 48 and 72 hours pi by performing two-way repeated-measures ANOVA [66] and tukey's HDS post-hoc comparisons (**Table S3 in Text S1**). This method was used before to analyze similar results [38]. A tukey's HDS post-hoc test was used to compare, once in Vero cells and once in C6/36 cells, the replicative fitness of the first passage for each virus (WT, Φnsp4 or Φnsp1 Φnsp4 Φenv virus) with that of the corresponding passaged virus. Comparison of the significance of the results from both methods showed that they gave very similar results (67/72 were concordant; **Table S3 in Text S1**). Infectious titres, viral RNA yields and relative fitness effect values were compared using a Student's *t* test. For all statistical tests used, all *p* values below 0.05 were considered significant.

## Supporting Information

Protocol S1 Cells and antibodies, construction of CHIKV infectious clones, plasmid transfection/virus stock production, plaque assay, tissue culture infectious dose 50 (TCID50) assay, real time RT-PCR assay, haemagglutination assay and quantification of intracellular viral proteins (ELISA method).
(PDF)

Text S1 Figure S1: Schematic representation of the CHIKV re-encoded viruses. Figure S2: Relationship between either infectious titres or viral RNA yields and the number of synonymous mutations in re-encoded region(s). Figure S3: Replication curves with WT passaged viruses. Figure S4: Replication curves with Φnsp4 passaged viruses. Figure S5: Replication curves with Φnsp1 Φnsp4 Φenv passaged viruses. Figure S6: Intra-population genetic diversity of CHIKV revealed using minimum spanning trees. Figure S7: Schematic representation of the CHIKV infectious clones (IC). Table S1: Genetic characteristics of the coding regions (concatenated ORFs) of the re-encoded viruses, the WT virus and 132 other CHIKVs extracted from GenBank. Table S2: Summary of Single cycle replication kinetics values at 8 and 14 hours pi. Table S3: Replicative fitness modifications observed during the passage experiments: comparison of the results from both analysis methods. Table S4: Mutations detected in the CHIKV consensus sequences during experimental passage. Table S5: Primers used for the sequencing of CHIKVs. Table S6: Primers and probes used for the real time RT-PCR assays.
(PDF)

Text S2 The re-encoded sequences, the list of the 132 CHIKV sequences extracted from GenBank, the nucleotide sequence of the synthetic RNA transcript used as standard for the universal real time RT-PCR assay and the amino-acid sequence of the recombinant protein used to immunized rabbits.
(PDF)
