# Supplementary material for: Random Codon Re-encoding Induces Stable Reduction of Replicative Fitness of Chikungunya Virus in Primate and Mosquito Cells
Source: PLoS Pathog. 2013 Feb 21;9(2):e1003172. doi: 10.1371/journal.ppat.1003172 (PMC3578757; doi:10.1371/journal.ppat.1003172)
Supplement: Protocol S1 — Cells and antibodies, construction of CHIKV infectious clones, plasmid transfection/virus stock production, plaque assay, tissue culture infectious dose 50 (TCID50) assay, real time RT-PCR assay, haemagglutination assay and quantification of intracellular viral proteins (ELISA method). (PDF) [file ppat.1003172.s001.pdf]

SUPPORTING INFORMATION: Protocol S1

Random codon re-encoding induces stable reduction of replicative fitness of Chikungunya virus in primate and mosquito cells

Antoine Nougairède, Lauriane De Fabritus, Fabien Aubry, Ernest A. Gould, Edward C. Holmes and Xavier De Lamballerie.

Cells and antibodies

African green monkey kidney (Vero) cells were grown at 37°C with 5% CO2 in a minimal essential medium (Invitrogen) with 7% heat-inactivated fetal bovine serum (FBS; Invitrogen) and 1% Penicillin/Streptomycin (PS; 5000U/ml and 5000µg/ml; Invitrogen). Human embryonic kidney 293 (HEK293) cells were grown at 37°C with 5% CO2 in Dulbecco's modified Eagles medium (Invitrogen) with 10% FBS and 1% PS. *A. albopictus* C6/36 cells were grown at 30°C in L-15 medium (Invitrogen) with 10% heat-inactivated FBS, 1% PS and 5% tryptose phosphate broth (29.5g/L; Sigma-Aldrich).

A CHIKV-specific immune human serum was used to perform the ELISA assay (see below). To decrease the concentration of non-specific molecules that react with HEK293 cell compounds, 40µl of serum was put in contact 16 hours with extracted HEK293 cells (cells obtained from one 150cm^2 flask culture, extracted using acetone) in a final volume of 400µl (diluents: 1% BSA; KPL). A recombinant protein (fusion between the C-terminal region of the nsP2 and the N-terminal region of the nsP3; **Text S2**), kindly provided by the AFMB laboratory (Architecture et Fonction des Macromolécules Biologiques, UMR 6098, Marseille France), was used to immunize two rabbits using standard methods (Rabbit Speedy 28-days immunization protocol, Eurogentec). Purified polyclonal antibodies (Affinity purification using a Sepharose matrix; Eurogentec) were used to perform the western blot analysis (see below).

Construction of CHIKV infectious clones (ICs)

We modified a previously described IC of the LR2006 strain [1] (GenBank accession EU224268) by replacing the origin of replication and the prokaryote promoter by a modified pBR322 origin and a promoter CMV (pCMV), respectively. BamHI and XhoI unique restriction sites were used to obtain an intermediate plasmid using standard molecular techniques which contained a new origin of replication (modified pBR322), the prokaryote

promoter CMV (pCMV) and the partial viral genome (from the first base to XhoI) (**Figure S7 in Text S1**). The partial viral genome (from XhoI to the end), the polyA tail and the hepatitis D ribozyme (HDR) followed by a Simian virus 40 (SV40) polyadenylation was synthesized (Genscript) and introduced into the intermediate construct using XhoI and AvrII unique restriction sites. Finally, unique restriction sites BamHI, AgeI and XhoI were used to introduce synonymous mutations into the genome (mutated cassettes were obtained by fusion of PCR products). A total of eight synonymous mutations were introduced to generate the required restriction sites or to eliminate undesirable restriction sites. The infectious clone obtained, which was considered the wild-type (WT), incorporated four new unique restriction sites (**Figure S7 in Text S1**).

All the re-encoded regions were synthesized (GenScript) and then inserted into ICs by digestion (BamHI/XmaI for Φnsp1, AgeI/ApaI for Φnsp4 and XhoI/AvrII for Φenv; NewEngland Biolabs), gel purification of digestion products (Qiagen), ligation (T4 DNA ligase; Invitrogen) and transformation into electrocompetent STBL4 cells (Invitrogen). Before their transfection, all the infectious clones were purified (0.22µm filtration) and their integrity was verified by restriction map and complete sequencing using a set of specific primer pairs.

Plasmid transfection and virus stock production

Each infectious clone was transfected into a 75 cm^2 culture flask containing subconfluent Vero cells (Fugene 6 transfection reagent; Roche); four hours after the addition of the transfection mixture, cells were washed twice (HBSS; Invitrogen) and 20 ml of medium was added. The cell supernatant was harvested when complete cytopathic effect (CPE) was evident (3-6 days depending on the virus), purified by centrifugation, aliquoted and stored at -80°C. Each virus was then passaged (called first passage in the study) in Vero cells at a estimated MOI of 0.5 in a 175 cm^2 culture flask: after incubation for 2 hours, the cells were washed twice (HBSS) and 50 ml of medium was added. The cell supernatant was harvested after 48 hours, clarified by centrifugation, aliquoted and stored at -80°C. These supernatants (virus stock) were used to perform single cycle replication kinetics, replication kinetics with low MOI, competition experiments, quantification of intracellular RNA and viral proteins and haemagglutination assays.

Plaque assay

Monolayers of Vero cells in 12-well culture plates were infected with 1 ml of virus stock (see above). After two hours, cells were washed (HBSS) and 2 ml of 0.9% agarose in culture medium was added. After an incubation of 72 hours, cells were fixed 4 hours with 10% formaldehyde and stained for 30 minutes with a 0.1% naphthalene black solution.

Tissue Culture Infectious Dose 50 (TCID50) assay

For each determination, a 96-well plate culture of confluent Vero cells was inoculated with 150µl/well of serial 10-fold dilutions of centrifugation clarified cell culture supernatants: each row included 6 wells of the dilution and two negative controls. The plates were incubated for 7 days and read for absence or presence of CPE in each well. The determination of the TCID50/ml was performed using the method of Reed and Muench [2]. When the value obtained with a sample was less than the detection threshold of the method ($10^{1.82}$ TCID50/ml), we performed another assay with two-fold, 20-fold and 200-fold dilutions (detection threshold: $10^{1.13}$ TCID50/ml). Values lower than this threshold were considered equal to $10^{1.13}$ TCID50/ml in the graphic presentations and were not taken into account in the statistical analyses. Assuming that the re-encoding and/or the experimental passages could modify significantly the appearance of CPE, we used a qRT-PCR assay (see below) as a sensitive indicator of the presence of infectious virus. This assay was performed for each virus (first passage and when available, 25th and 50th passages). For all the viruses, CPE positive wells were positive in qRT-PCR with a threshold cycle lower than 16 while those that failed to produce CPE were negative or positive with a threshold cycle >35, the value expected after the dilution of the initial RNA yields.

Real time RT-PCR assay

A fragment of 179 nt located in the nsP2 region (nucleotide position 2631 to 2809) was used to detect all the CHIKVs (universal assay), re-encoded or not. Another fragment of 168 nt located in the nsP4 region (nucleotide position 6804 to 6971) was used to analyze cell supernatants from competition experiments: two sets of primers and probes allowed us to detect specifically either the viruses re-encoded in the nsP4 region or the viruses without modification in the same region. Primer and probe sequences are detailed in **Table S6 in Text S1**.

The mixture (final volume: 25µl) contained a standard quantity of 2X PCR Master Mix and SuperScript III RT/Platinum Taq Mix (Invitrogen), 0.4µM of each primer and either 4µl of diluted (1/5) extracted nucleic acids or 4µl of water (negative control). Assays were performed on Mx3005p thermocycler (Stratagene) with the following conditions: 50°C for 15 min, 95°C for 2 min, followed by 45 cycles of 95°C for 15 s, 60°C for 40 s. Data collection occurred during the 60°C step.

The amount of viral RNA was calculated from standard curves using a synthetic RNA transcript (**Text S2**) for the universal assay whilst viral nucleic acids from cell supernatants of cultured WT virus or Φnsp4 virus were used as standard for both specific real time assays (five nucleic acid extracts were pooled and 10µl-aliquots were stored at -80°C). Values of the quantity of viral RNA/ml for each standard used for both specific real time RT-PCR assays were obtained using the universal assay with the *in vitro* RNA transcript as a standard. The

standard curve generated for all these assays had values of coefficient of determination (R^2) >0.97 and the amplification efficiency was between 94% and 104%.

Haemagglutination assay

An estimated MOI of 5 was used to infect with virus stock (see above) a 25cm^2 culture flask of confluent Vero or C6/36 cells. Cells were washed twice (HBSS) 30 minutes after the infection and 8 ml of medium without FBS was added. 2 ml of cell supernatant was sampled at 16 hours pi. Sampled supernatants were clarified by centrifugation, aliquoted and stored at -80°C. They were then analysed using a TCID50 assay (see above), a real time RT-PCR assay (cf. supra see above) and a haemagglutination titration assay was performed using standard methods [3]: twofold serial dilutions of cell supernatant on U-bottom microplates were prepared in 0.4% bovine albumin/borate saline pH 9.0 solution (final volume: 35 µl per well). Thirty-five microliters of pre-diluted goose red blood cells (1/150 using the final pH 6.0 adjusting diluents) were added, the mixture was homogenized, incubated for 45 min at room temperature and then read using four scoring symbols: ++ for complete haemagglutination, + for partial haemagglutination, +/− for trace haemagglutination and − for negative haemagglutination. The haemagglutination titre was the reciprocal of the highest dilution in which + was observed.

Quantification of intracellular viral proteins: ELISA method

A global estimated MOI of 5 was used to infect confluent 12 well-plates of HEK293 cells with virus stock (see above). Cells were washed once (HBSS) 30 minutes after the infection and 2 ml of media was added. At 8 hours pi, absence of cytopathic effect was checked, culture supernatants were discarded and cells were washed once (HBSS). All experiments were performed in triplicate.

Cells were mechanically harvested using a cell scraper, resuspended in 800µL of PBS, vortexed and disrupted by sonication (30 seconds at 20 KHz, Misonix Sonicator XL). After purification by centrifugation, cell extracts were diluted (1/5 in PBS, 100µl) and coated overnight at 4°C on 96-well polystyrene plates. Then the plates were blocked with 350µl of 1% BSA (KPL) for 15 minutes at room temperature. Following a brief wash (PBS), plates were incubated 1 hour at room temperature with 100µl of pre-treated CHIKV-specific immune human serum (cf. supra), washed three times (PBS tween-20 0.04%), incubated 1 hour at room temperature with 100µl of the corresponding HRP-conjugated secondary antibody, washed three times (PBS tween-20 0.04%) and incubated 15 minutes at room temperature with 100µl of substrate (SureBlue, KPL). The reaction was blocked with 100µl of 2N sulphuric acid. Plates were read using a microplate reader (Sunrise, Tecan) at a wavelength of 450-nm.

References

1. Tsetsarkin K, Higgs S, McGee CE, De Lamballerie X, Charrel RN, et al. (2006) Infectious clones of Chikungunya virus (La Reunion isolate) for vector competence studies. Vector Borne Zoonotic Dis 6: 325-337.

2. Reed LJ, Muench H (1938) A simple method of estimating fifty per cent endpoints. Am J Hyg 27: 493-497.

3. Clarke DH, Casals J (1958) Techniques for hemagglutination and hemagglutination-inhibition with arthropod-borne viruses. Am J Trop Med Hyg 7: 561-573.
