# Supplementary material for: Random Codon Re-encoding Induces Stable Reduction of Replicative Fitness of Chikungunya Virus in Primate and Mosquito Cells
Source: PLoS Pathog. 2013 Feb 21;9(2):e1003172. doi: 10.1371/journal.ppat.1003172 (PMC3578757; doi:10.1371/journal.ppat.1003172)
Supplement: Text S1 — Figure S1: Schematic representation of the CHIKV re-encoded viruses. Figure S2: Relationship between either infectious titres or viral RNA yields and the number of synonymous mutations in re-encoded region(s). Figure S3: Replication curves with WT passaged viruses. Figure S4: Replication curves with Φnsp4 passaged viruses. Figure S5: Replication curves with Φnsp1 Φnsp4 Φenv passaged viruses. Figure S6: Intra-population genetic diversity of CHIKV revealed using minimum spanning trees. Figure S7: Schematic representation of the CHIKV infectious clones (IC). Table S1: Genetic characteristics of the coding regions (concatenated ORFs) of the re-encoded viruses, the WT virus and 132 other CHIKVs extracted from GenBank. Table S2: Summary of Single cycle replication kinetics values at 8 and 14 hours pi. Table S3: Replicative fitness modifications observed during the passage experiments: comparison of the results from both analysis methods. Table S4: Mutations detected in the CHIKV consensus sequences during experimental passage. Table S5: Primers used for the sequencing of CHIKVs. Table S6: Primers and probes used for the real time RT-PCR assays. (PDF) [file ppat.1003172.s002.pdf]

SUPPORTING INFORMATION: Text S1

Random codon re-encoding induces stable reduction of replicative fitness of Chikungunya virus in primate and mosquito cells

Antoine Nougairède, Lauriane De Fabritus, Fabien Aubry, Ernest A. Gould, Edward C. Holmes and Xavier De Lamballerie.

Table of Contents

Supplementary Figures

Figure S1: Schematic representation of the CHIKV re-encoded viruses 2

Figure S2: Relationship between either infectious titres or viral RNA yields and the number of synonymous mutations in re-encoded region(s) 3

Figure S3: Replication curves with WT passaged viruses 4

Figure S4: Replication curves with Φnsp4 passaged viruses 5

Figure S5: Replication curves with Φnsp1 Φnsp4 Φenv passaged viruses 6

Figure S6: Minimum spanning trees 7

Figure S7: Schematic representation of the CHIKV infectious clone 10

Supplementary Tables

Table S1: Genetic characteristics of the coding regions of the re-encoded viruses, the WT virus and 132 other CHIKVs extracted from GenBank 11

Table S2: Summary of Single cycle replication kinetics values at 8-14 hours pi 12

Table S3: Replicative fitness modifications observed during the passage experiments: comparison of the results from both analysis methods 14

Table S4: Mutations detected in consensus sequences during the passages 15

Table S5: Primers used in this study for the sequencing of CHIKVs 17

Table S6: Primers and probes used for the real time RT-PCR assays 19

References 20

Supplementary Figures

Figure S1: Schematic representation of the CHIKV re-encoded viruses.

From top to bottom: Nucleotide scale bar; schematic representation of the CHIKV complete genome with coding regions (grey rectangles), non-coding (black rectangles) and the polyA tail.

Reencoded regions are represented in dark grey.

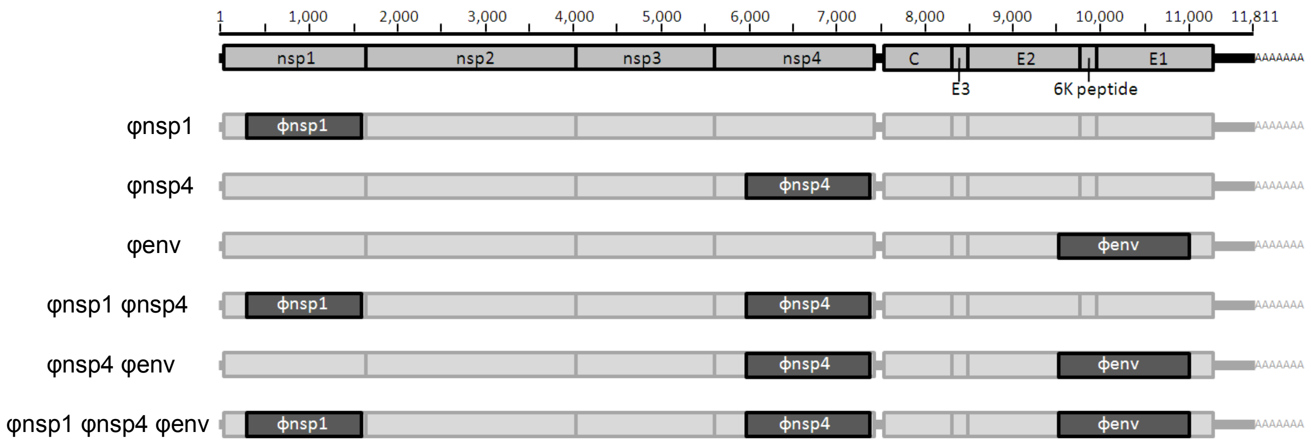

Figure S2: Relationship between either infectious titres or viral RNA yields and the number of synonymous mutations in re-encoded region(s).

Infectious titres are represented by the results of the TCID50 assay and viral RNA yields by the results of the real time RT-PCR assay, both performed using cell supernatants of single cycle replication kinetics at 14 hours pi. Results are the mean and standard deviation from three independent experiments.

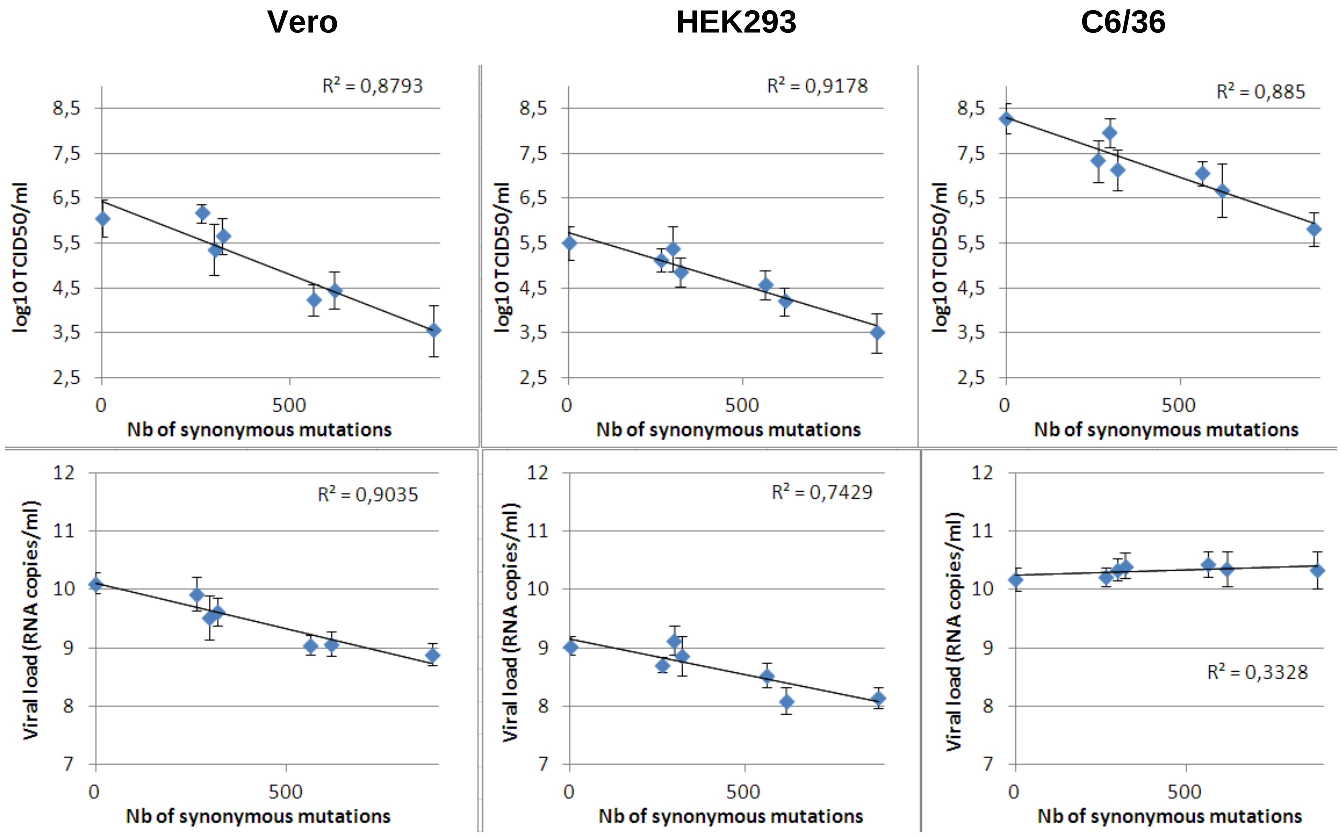

Figure S3: Replication curves with WT passaged viruses.

Replication kinetic experiments were performed in Vero cells (**a-c**) and C6/36 cells (**d-f**). Panels **a** and **d** represent viruses serially passaged in Vero cells, **b** and **e** viruses serially passaged in C6/36 and **c** and **f** viruses alternatively passaged. Analysis of experiments in Vero cells (ANOVA) revealed a significant effect of passage (df=12 ; p<0.001), of day (df=2 ; p<0.001) and of interaction between both factors (df=24 ; p<0.001). Analysis of experiments in C6/36 cells (ANOVA) revealed no significant effect of passages (df=12 ; p=0.125), significant effect of day (df=2 ; p<0.001) and significant effect of interaction between both factors (df=24 ; p=0.027).

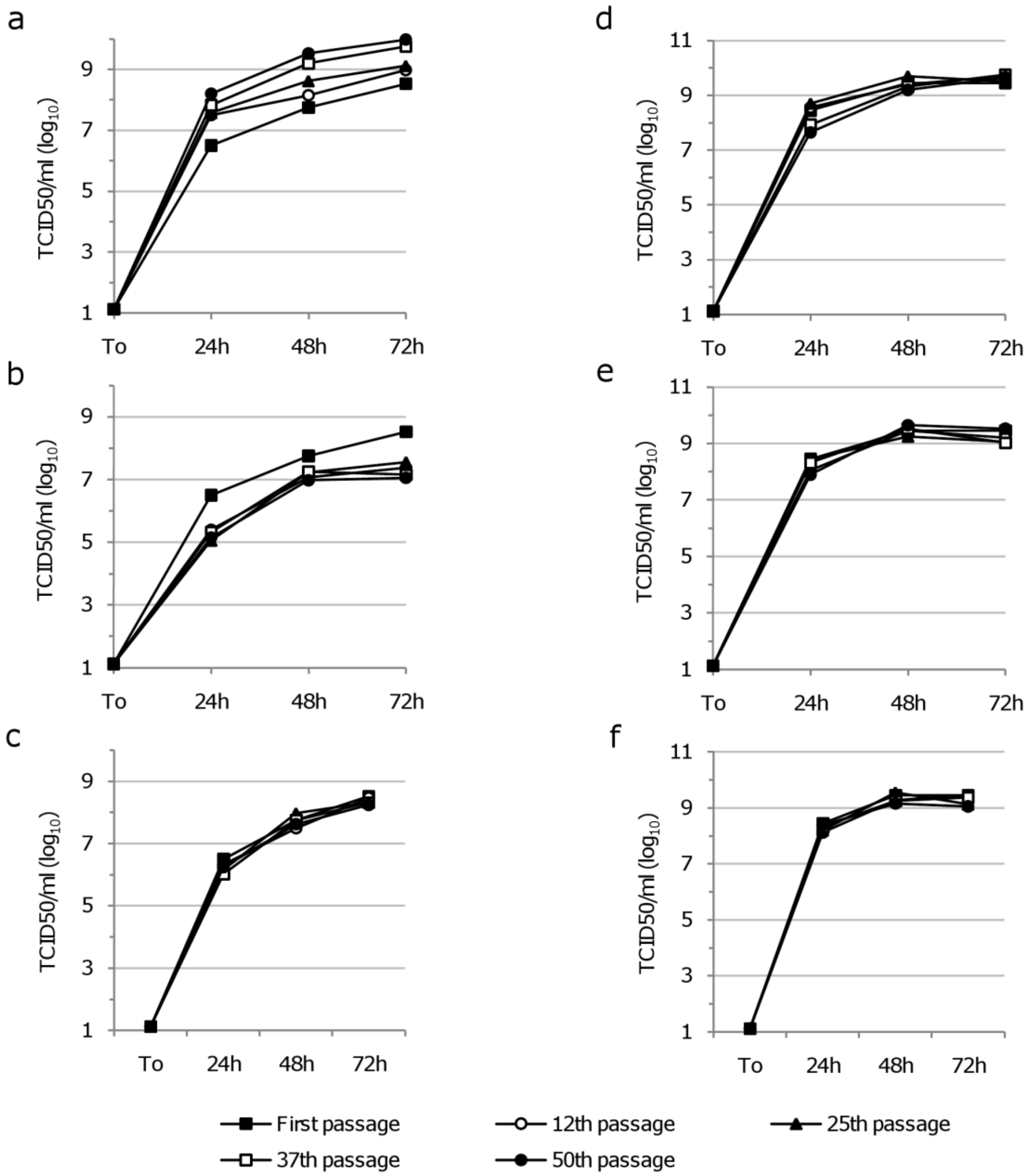

Figure S4: Replication curves with Φnsp4 passaged viruses.

Replication kinetics experiments were performed in Vero cells (**a-c**) and C6/36 cells (**d-f**). Panels **a** and **d** represent viruses serially passaged in Vero cells, **b** and **e** viruses serially passaged in C6/36 and **c** and **f** viruses alternately passaged. Analysis of experiments in Vero and C6/36 cells (ANOVA) revealed a significant effect of passages (df=12 ; p<0.001), of day (df=2 ; p<0.001) and of interaction between both factors (df=24 ; p<0.001 and p=0.001, respectively).

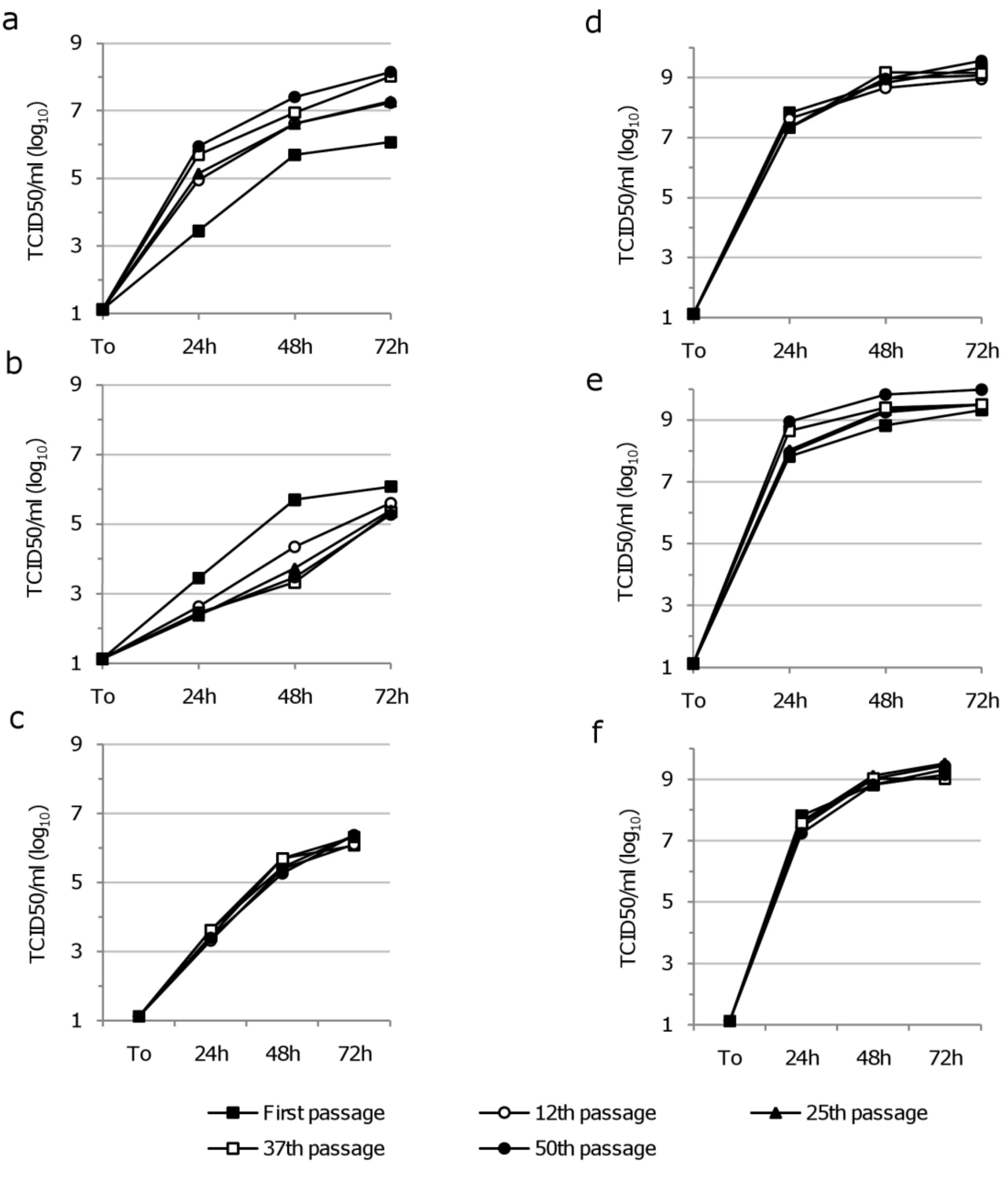

Figure S5: Replication curves with Φnsp1 Φnsp4 Φenv passaged viruses.

Replication kinetics experiments were performed in Vero cells (**a-c**) and C6/36 cells (**d-f**). Panels **a** and **d** represent viruses serially passaged in Vero cells, **b** and **e** viruses serially passaged in C6/36 and **c** and **f** viruses alternately passaged. Analysis of experiments in Vero and C6/36 cells (ANOVA) revealed a significant effect of passages (df=12 ; p<0.001), of day (df=2 ; p<0.001) and of interaction between both factors (df=24 ; p<0.001).

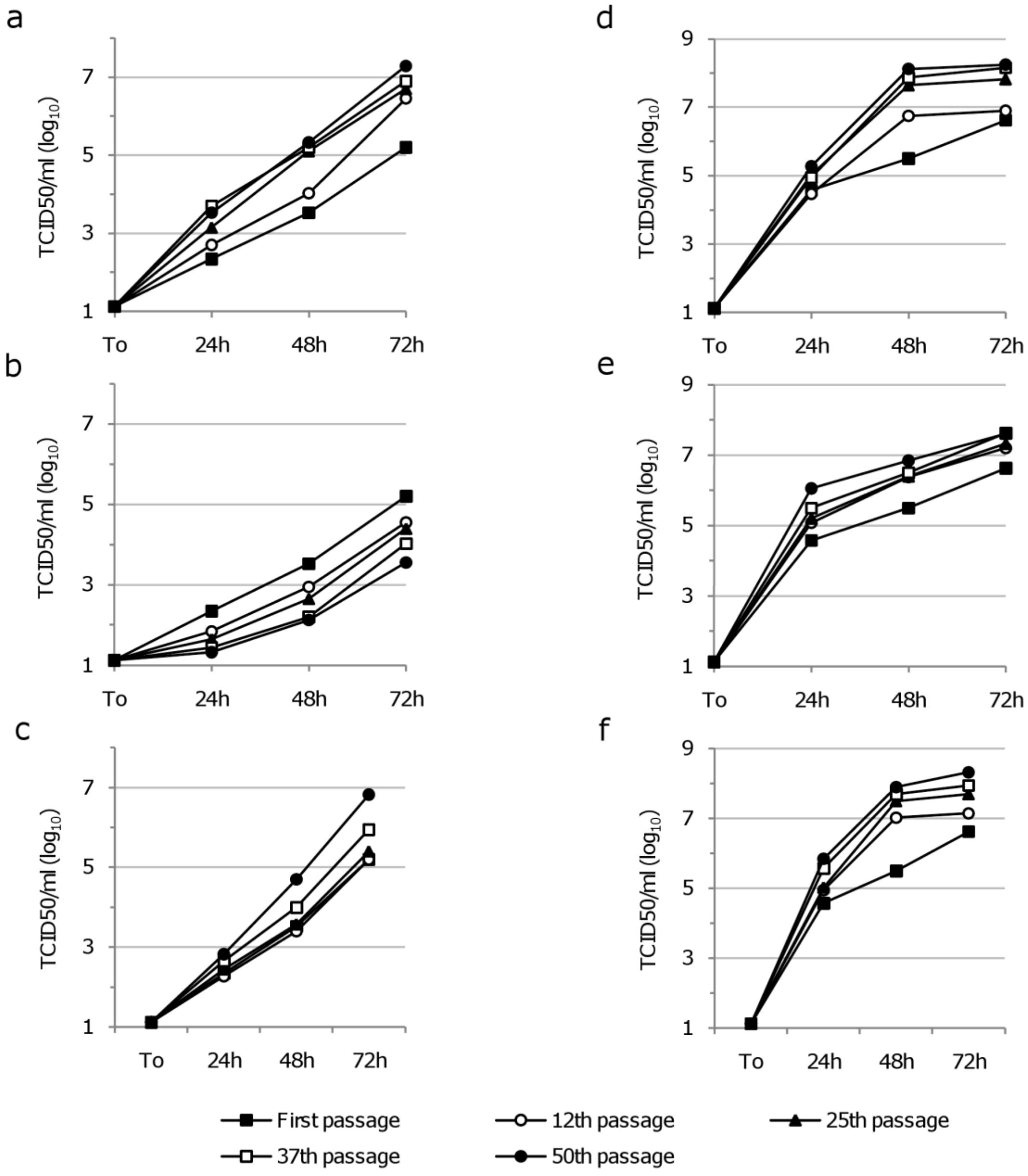

Figure S6: Intra-population genetic diversity of CHIKV revealed using minimum spanning trees.

We used minimum spanning trees to explore the dynamics of intra-population genetic diversity of the three regions analyzed (nsP2/nsP3, E3/E2 and E1). Each panel represents all the clones sequenced in one region of one virus passage following one method: (**a**) E3/E2 region of the Reenc 1 virus passaged alternately, (**b**) nsP2/nsP3 region of the Φnsp4 virus serially passaged in c6/36 cells, (**c**) nsP2/nsP3 region of the Φnsp1 Φnsp4 Φenv virus serially passaged in c6/36 cells, (**d**) nsP2/nsP3 region of the Φnsp1 Φnsp4 Φenv virus serially passaged in Vero cells, (**e**) E3/E2 region of the WT virus serially passaged in Vero cells, (**f**) E1 region of the Φnsp1 Φnsp4 Φenv virus serially passaged in c6/36 cells, (**g**) nsP2/nsP3 region of the Φnsp1 Φnsp4 Φenv virus alternately passaged, (**h**) E3/E2 region of the Φnsp1 Φnsp4 Φenv virus serially passaged in Vero cells, (**i**) E3/E2 region of the WT virus serially passaged in C6/36 cells, (**j**) E3/E2 region of the Φnsp1 Φnsp4 Φenv virus serially passaged in C6/36 cells, (**k**) E3/E2 region of the Φnsp4 virus serially passaged in Vero cells, (**l**) E1 region of the Φnsp1 Φnsp4 Φenv virus alternately passaged and (**m**) E3/E2 region of the WT virus alternately passaged.

Each circle represents one variant and its size corresponds to the number of clones with the same nt sequence. The original sequence is represented by the biggest circle except in panel (**f**) where it is the circle at the top. Mutation positions are indicated in each branch. For the point mutations, the nt present in each viral population is shown. For the deletions which were considered as a unique event, the word 'Del' indicates that they are present in the nearest viral population. For the two deletions of 9 nt found in Φnsp4 virus serially passaged in C6/36 cells at nt positions 4139/47 and 4164/74 (panel **b**), (i) no modification compared to the original sequence are represented by C and G respectively, and (ii) A for the second deletion means the presence of the 4167g>a mutation. For the two deletions of 6 nt found in the viruses serially passaged in C6/36 cells at nt positions 8556/61 (panel **j**) and 8563/8 (panel **i** and **j**), (i) no modification compared to the original sequence are represented by U, and (ii) C for the second deletion means the presence of the 8566u>c mutation.

Figure S6 (**continued**)**:** Intra-population genetic diversity of CHIKV revealed using minimum spanning trees.

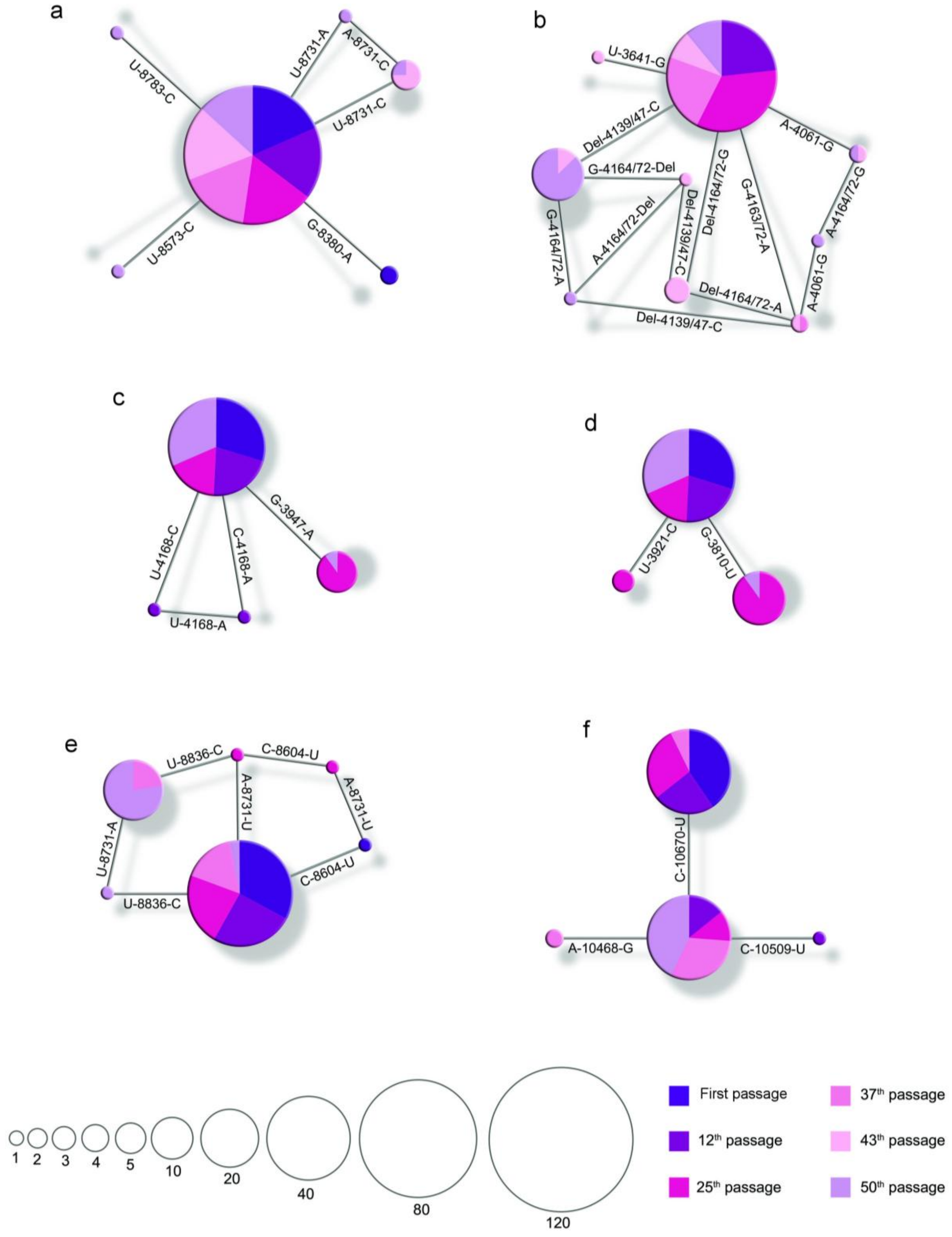

Figure S6 (**continued**)**:** Intra-population genetic diversity of CHIKV revealed using minimum spanning trees.

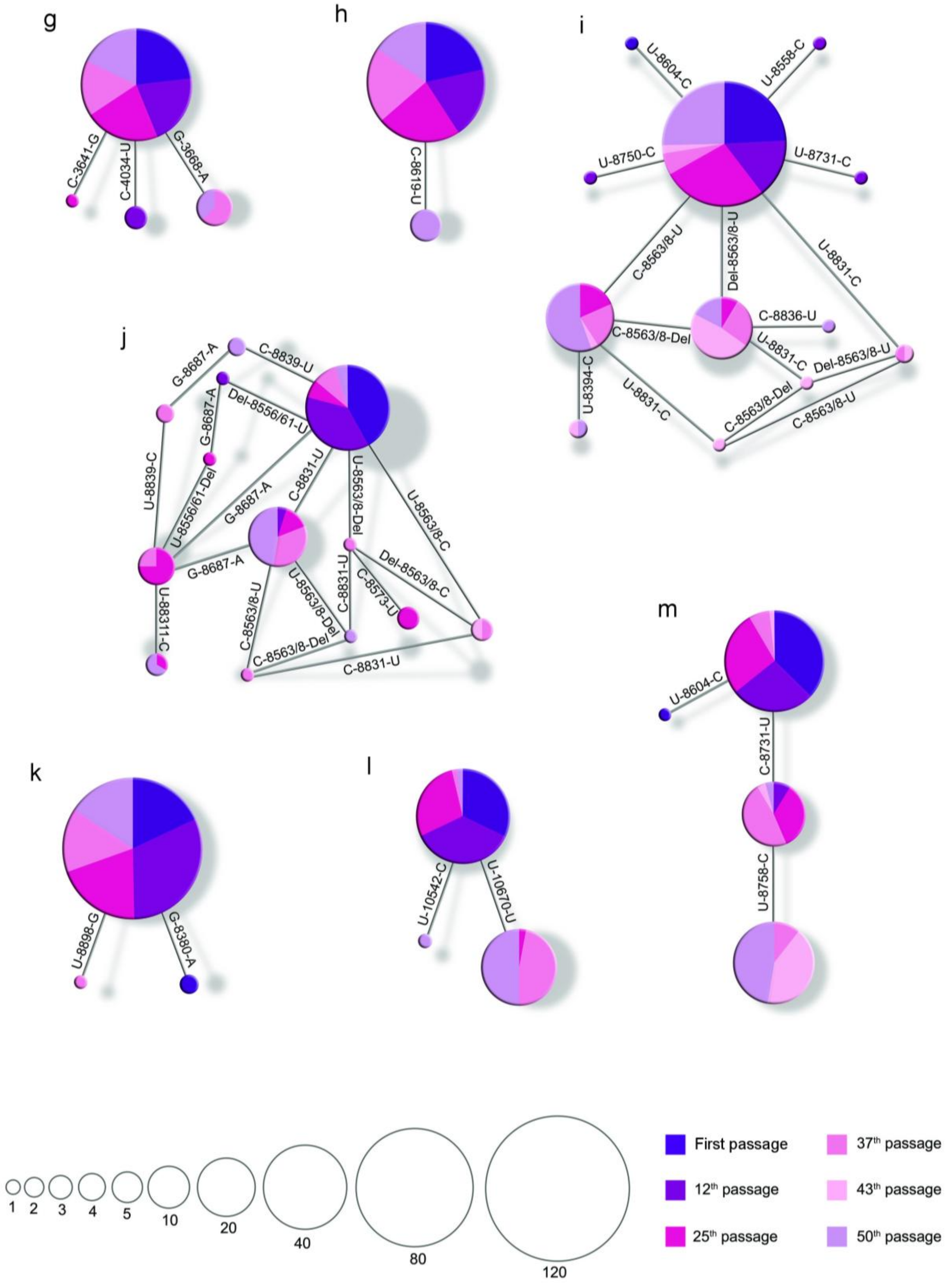

Figure S7: Schematic representation of the CHIKV infectious clones (IC).

Coding (grey rectangles) and non-coding (black rectangles) regions which represent the complete genome of the CHIKV were flanked in 5' and 3' by the CMV promoter (pCMV) and the HRD-SV40-pAs (hepatitis delta ribozyme followed by the simian virus 40 polyadenylation signal). All these regions were inserted into a modified pBR322 plasmid.

Unique restriction sites are represented in the figure. Finally, 8 synonymous mutations (black arrows; the asterisks means that two mutations located close to one another were introduced) allowed us to introduce 4 new unique restriction sites (sites underlined) when compared to the previously described IC of the LR2006 strain [1] (GenBank accession EU224268).

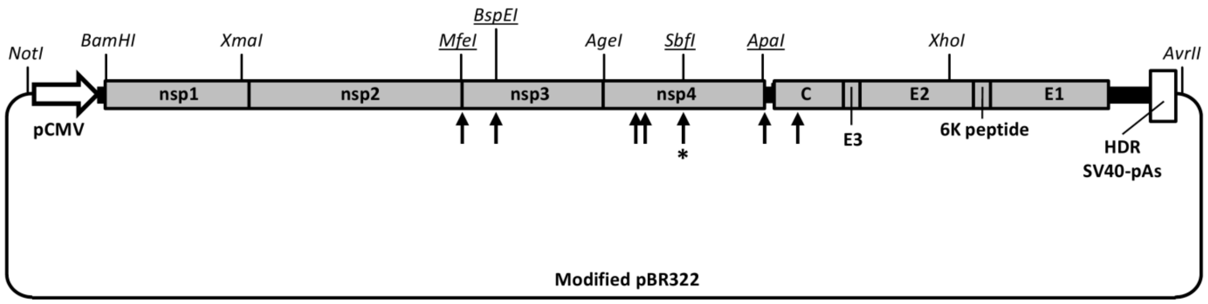

Supplementary Tables

Table S1: Genetic characteristics of the coding regions (concatenated ORFs) of the re-encoded viruses, the WT virus and 132 other CHIKVs extracted from GenBank (see above).

The %G+C and the number of CpG and UpA dinucleotides were calculated using the Dambe software [2]. Codon usage was measured using the effective number of codons prime (Nc') [3] which gives a value ranging from 20 (only one codon used for each amino acid) to 61 (random codon usage for each amino acid) and accounting for background nucleotide composition. The 132 CHIKV sequences were those extracted from GenBank (**Text S2**).

Virus	Length of codon replacement interval (nt)	No. of mutations compared with WT	Concatenate ORFs			
			Nc'	%G+C	No. of dinucleotides	
					CpG	UpA
**WT**	-	-	57,7	51,0	599	558
**Φnsp1**	1302	264	58,4	50,6	596	575
**Φnsp4**	1410	298	58,3	50,8	608	572
**Φenv**	1500	320	58,2	50,7	595	570
**Φnsp1 Φnsp4**	2712	562	58,9	50,5	605	589
**Φnsp4 Φenv**	2910	618	58,8	50,3	604	584
**Φnsp1 Φnsp4 Φenv**	4212	882	59,2	50,0	601	601
**132 CHIKV sequences [min ; max]**	-	-	[56,6 ; 58,2]	[50,5 ; 51,3]	[577 ; 615]	[505 ; 591]

Table S2: Summary of Single cycle replication kinetics values at 8 and 14 hours pi.

The term infectivity means infectivity of viral particles (*i.e.*, the ratio of the number of infectious particles [calculated here using a TCID50 assay] over the number of viral particles [calculated here using a quantitative PCR assay]). The value represented here was normalized to the WT value (allowing direct comparison between the infectivity of each re-encoded virus and that of the WT virus).

The *p* value is that of a student's *t* test comparing each re-encoded virus with the WT virus.

¶ means significant result (p value < 0.05).

Table S2 (continued): Summary of Single cycle replication kinetics values at 8 and 14 hours pi.

cells	hours pi	virus	log_{10} TCID50/ml		log_{10} RNA copies/ml		Infectivity
			mean +/- SD	*p* value	mean +/- SD	*p* value	
Vero	8	WT	3.90 +/- 0.36		7.98 +/- 0.29		1
		ϕnsp1	3.71 +/- 0.30	0.259	8.35 +/- 0.30	0.094	1/4
		ϕnsp4	4.00 +/- 0.52	0.399	8.15 +/- 0.26	0.232	1
		ϕenv	3.40 +/- 0.36	0.082	7.70 +/- 0.17	0.122	1/2
		ϕnsp1 ϕnsp4	3.33 +/- 0.35	0.060	7.93 +/- 0.18	0.424	1/3
		ϕnsp4 ϕenv	2.98 +/- 0.25	0.011 ¶	7.74 +/- 0.08	0.133	1/5
		ϕnsp1 ϕnsp4 ϕenv	2.75 +/- 0.41	0.011 ¶	7.85 +/- 0.24	0.304	1/10
	14	WT	6.05 +/- 0.41		10.11 +/- 0.18		1
		ϕnsp1	6.17 +/- 0.21	0.341	9.92 +/- 0.29	0.191	1
		ϕnsp4	5.35 +/- 0.58	0.081	9.52 +/- 0.38	0.034 ¶	1/2
		ϕenv	5.65 +/- 0.41	0.149	9.62 +/- 0.24	0.024 ¶	1
		ϕnsp1 ϕnsp4	4.23 +/- 0.35	0.002 ¶	9.05 +/- 0.16	<0.001 ¶	1/9
		ϕnsp4 ϕenv	4.45 +/- 0.41	0.004 ¶	9.07 +/- 0.20	0.001 ¶	1/5
		ϕnsp1 ϕnsp4 ϕenv	3.55 +/- 0.56	0.002 ¶	8.89 +/- 0.19	<0.001 ¶	1/23
HEK293	8	WT	3.75 +/- 0.40		7.30 +/- 0.23		1
		ϕnsp1	3.45 +/- 0.51	0.233	7.22 +/- 0.12	0.293	1
		ϕnsp4	3.85 +/- 0.25	0.365	7.67 +/- 0.25	0.068	1/2
		ϕenv	2.85 +/- 0.48	0.033 ¶	7.47 +/- 0.28	0.234	1/9
		ϕnsp1 ϕnsp4	3.05 +/- 0.31	0.037 ¶	7.48 +/- 0.16	0.170	1/8
		ϕnsp4 ϕenv	2.93 +/- 0.33	0.025	7.16 +/- 0.18	0.224	1/5
		ϕnsp1 ϕnsp4 ϕenv	2.75 +/- 0.39	0.018 ¶	7.22 +/- 0.20	0.328	1/8
	14	WT	5.50 +/- 0.38		9.03 +/- 0.16		1
		ϕnsp1	5.13 +/- 0.25	0.113	8.72 +/- 0.13	0.027 ¶	1
		ϕnsp4	5.38 +/- 0.51	0.374	9.13 +/- 0.26	0.332	1/2
		ϕenv	4.85 +/- 0.31	0.042 ¶	8.86 +/- 0.34	0.235	1/3
		ϕnsp1 ϕnsp4	4.58 +/- 0.33	0.016 ¶	8.53 +/- 0.20	0.014 ¶	1/3
		ϕnsp4 ϕenv	4.20 +/- 0.31	0.005 ¶	8.09 +/- 0.23	0.002 ¶	1/2
		ϕnsp1 ϕnsp4 ϕenv	3.50 +/- 0.44	0.002 ¶	8.15 +/- 0.18	0.002 ¶	1/12
C6/36	8	WT	5.75 +/- 0.36		8.95 +/- 0.24		1
		ϕnsp1	5.70 +/- 0.13	0.416	9.03 +/- 0.13	0.329	1
		ϕnsp4	6.13 +/- 0.29	0.116	8.73 +/- 0.17	0.127	3
		ϕenv	5.68 +/- 0.28	0.396	8.59 +/- 0.21	0.058	2
		ϕnsp1 ϕnsp4	6.05 +/- 0.28	0.159	9.06 +/- 0.19	0.285	1
		ϕnsp4 ϕenv	5.80 +/- 0.65	0.457	9.06 +/- 0.20	0.296	1
		ϕnsp1 ϕnsp4 ϕenv	4.15 +/- 0.35	0.003 ¶	9.00 +/- 0.10	0.376	1/45
	14	WT	8.28 +/- 0.34		10.18 +/- 0.19		1
		ϕnsp1	7.33 +/- 0.46	0.022 ¶	10.22 +/- 0.16	0.399	1/9
		ϕnsp4	7.95 +/- 0.33	0.148	10.34 +/- 0.19	0.173	1/3
		ϕenv	7.13 +/- 0.46	0.012 ¶	10.41 +/- 0.21	0.115	1/23
		ϕnsp1 ϕnsp4	7.05 +/- 0.26	0.004 ¶	10.43 +/- 0.22	0.101	1/31
		ϕnsp4 ϕenv	6.68 +/- 0.59	0.007 ¶	10.35 +/- 0.30	0.219	1/53
		ϕnsp1 ϕnsp4 ϕenv	5.80 +/- 0.38	<0.001 ¶	10.34 +/- 0.32	0.250	1/420

Table S3: Replicative fitness modifications observed during the passage experiments: comparison of the results from both analysis methods.

To study the replicative fitness evolution over the passages in response to codon re-encoding, we performed replicative kinetics at the 1st, 12th, 25th, 37th and 50th passages of each virus in Vero and C6/36 cells (See above **Figure S2-4**). We first measured the viral growth rate at 24 hours pi, based on TCID50 values. The corresponding relative fitness effect values are detailed in **Figure 7**. We then performed a global analysis of TCID50 values at 24, 48 and 72 hours pi by performing two-way repeated-measures ANOVA and tukey's HDS post-hoc comparisons. Significant results of both analyses are summarized here.

Symbol legend: Significant (p<0.05) fitness enhancement (↗) or reduction (↘) and no significant fitness modification (-) in comparison with the first passage of the corresponding virus. The first symbol indicates the results obtained with relative fitness effect values and the second those obtained using the Tukey HSD post-hoc comparison. Discordant results are emboldened and cells are shaded.

Virus	Passage method	Passage no. tested in Vero cells				Passage no. tested in C6/36 cells			
		12	25	37	50	12	25	37	50
WT	Vero	↗ ↗	↗ ↗	↗ ↗	↗ ↗	- -	- -	- -	- -
	C6/36	↘ ↘	↘ ↘	↘ ↘	↘ ↘	- -	- -	- -	- -
	Alternate	- -	- -	**↘ -**	- -	- -	- -	- -	- -
Φnsp4	Vero	↗ ↗	↗ ↗	↗ ↗	↗ ↗	- -	**↘ -**	- -	- -
	C6/36	↘ ↘	↘ ↘	↘ ↘	↘ ↘	- -	**- ↗**	↗ ↗	↗ ↗
	Alternate	- -	- -	- -	- -	- -	- -	- -	**↘ -**
Φnsp1 Φnsp4 Φenv	Vero	↗ ↗	↗ ↗	↗ ↗	↗ ↗	**- ↗**	↗ ↗	↗ ↗	↗ ↗
	C6/36	↘ ↘	↘ ↘	↘ ↘	↘ ↘	↗ ↗	↗ ↗	↗ ↗	↗ ↗
	Alternate	- -	- -	↗ ↗	↗ ↗	↗ ↗	↗ ↗	↗ ↗	↗ ↗

Table S4: Mutations detected in the CHIKV consensus sequences during experimental passage.

REENC1 means Φnsp4 virus and REENC3 means Φnsp1 Φnsp4 Φenv virus. 'Del' means deletion, and the last 9 columns represent the results of the complete genome consensus sequencing, in which 'P' represents the passage number. All the mixed viral populations (when double peaks were observed on the sequencing chromatograms) were represented by ≈, > and < in the table.

Virus	Passage method	Nt position	Region	Nt change	AA change	P1	P6	P12	P18	P25	P31	P37	P43	P50
**WT**	**Vero**	176	nsP1	C→U	P→S	C	C	C	C	C	C	C	C	C≈U
	**Vero**	1226	nsP1	C→A	L→I	C	C	A	A	A	A	A	A	A
	**Vero**	4289	nsP3	A→G	N→D	A	A	A	A	A	A	A	A	A≈G
	**Vero**	4456	nsP3	C→U	-	C	C	C	C	C	C	C≈U	C<U	C<U
	**Vero**	5446	nsP3	C→U	-	C	U	U	U	U	U	U	U	U
	**Vero**	5819	nsP4	C→U	L→F	C	C	C	C	C	C	C	C<U	C<U
	**Vero**	6426	nsP4	C→U	A→V	C	C	C	C	C	C≈U	U	U	U
	**Vero**	8731	E2	U→A	W→R	U	U	U	U	U	U	U≈A	U<A	U<A
	**Vero**	8836	E2	C→U	H→Y	C	C	C	C	C	C>U	C≈U	C<U	U
	**Vero**	9043	E2	A→G	E→K	A	G	G	G	G	G	G	G	G
	**C6/36**	8563-8	E2	Del-GUCUAU	Del-VY	-	-	-	-	-	>Del	≈Del	<Del	-
	**C6/36**	8566	E2	U→C	Y→H	U	U	U	U	U>C	U≈C	U≈C	U≈C	U≈C
	**C6/36**	10670	E1	U→C	V→A	U	U	U	U<C	U≈C	U>C	U>C	U>C	U>C
	**Vero/C636**	4043	nsP2	G→A	A→T	G	G	G	G	G	G	G≈A	A	A
	**Vero/C636**	5224	nsP3	C→U	-	C	C	C	C	C	C	C	C>U	C≈U
	**Vero/C636**	6426	nsP4	C→U	A→V	C	C	C	C	C	C	C	C	C≈U
	**Vero/C636**	8731	E2	U→C	W→R	U	U	U	U	U>C	U≈C	U≈C	C	C
	**Vero/C636**	8758	E2	C→U	H→Y	C	C	C	C	C	C	C>U	U	U
	**Vero/C636**	10670	E1	U→C	V→A	U	U	U	U	U	U	U	U	U≈C
**REENC1**	**Vero**	202	nsP1	G→C	-	G	G	G	G	G	G	G	G<C	G<C
	**Vero**	1903	nsP2	C→U	-	C	C≈U	U	U	U	U	U	U	U
	**Vero**	4636	nsP3	A→C	K→N	A	A	A	A	A	A>C	A≈C	A<C	C
	**Vero**	5486	nsP3	U→C	S→P	U	U	U	U	U	U	U	U≈C	U<C
	**Vero**	6330	nsP4	U→C	V→A	U	U	U	U	U	U	U≈C	C	C
	**Vero**	6971	nsP4	U→C	-	U	U	U	U>C	C	C	C	C	C
	**Vero**	8139	C	C→U	-	C	C	C	U	U	U	U	U	U
	**Vero**	9580	E2	A→G	T→A	A	G	G	G	G	G	G	G	G
	**C6/36**	4139-47	nsP3	Del-GCCGCUAAC	Del-AAN	-	-	-	-	-	-	-	≈Del	<Del
	**C6/36**	6670	nsP4	C→A	N→K	C	C	C	C	C	C	C	C>A	C<A
	**C6/36**	8563-8	E2	Del-GUCUAU	Del-VY	-	-	-	-	-	>Del	≈Del	<Del	-
	**C6/36**	8566	E2	U→C/A	Y→H	U	U	U	U	U	U>C	U≈C/A	U<C	U<C
	**C6/36**	10574	E1	C→U	A→V	C	C	C	C	C	C	C	C≈U	C<U
	**C6/36**	10670	E1	U→C	V→A	U	U	U	U	U>C	U>C	U>C	U≈C	U>C
	**Vero/C636**	3218	nsP2	U→G	S→A	U	U	U	U	U≈G	U≈G	U≈G	U≈G	U<G
	**Vero/C636**	4167	nsP3	G→A	G→D	G	G	G	G	G	G	G≈A	G≈A	G>A
	**Vero/C636**	4299	nsP3	A→G	N→S	A	A	A	A	A	A	A	A≈G	A≈G
	**Vero/C636**	5376	nsP3	U→C	L→P	U	U	U	U	U	U≈C	U≈C	U≈C	U<C
	**Vero/C636**	7779	C	G→A	-	G	G	G	G	G	G>A	G≈A	G≈A	G<A
	**Vero/C636**	9083	E2	U→C	M→T	U	U	U	U	U	U>C	U≈C	U≈C	U<C
	**Vero/C636**	10670	E1	U→C	V→A	U	U	U	U	U	U	U<C	U≈C	U>C

Table S4 (continued): Mutations detected in the CHIKV consensus sequences during experimental passage.

Virus	Passage method	Nt position	Region	Nt change	AA change	P1	P6	P12	P18	P25	P31	P37	P43	P50
**REENC3**	**Vero**	22	5'UTR	A→G	-	A	A<G	G	G	G	G	G	G	G
	**Vero**	66	5'UTR	A→G	-	A	G	G	G	G	G	G	G	G
	**Vero**	822	nsP1	C→U	P→L	C	C	C	C	C	C>U	C>U	C≈U	C≈U
	**Vero**	1703	nsP2	G→A	G→R	G	G	G	G	G	G≈A	G<A	G<A	G<A
	**Vero**	3810	nsP2	G→U	G→V	G	G	G	G>U	G≈U	G≈U	G≈U	G≈U	G≈U
	**Vero**	5601	nsP3	C→U	T→I	C	C	C≈U	C<U	U	U	U	U	U
	**Vero**	6717	nsP4	A→C	E→A	A	A	A	A	A	A	A	A>C	A≈C
	**Vero**	6761	nsP4	A→G	T→A	A	A	A<G	G	G	G	G	G	G
	**Vero**	7742	C	A→G	Q→R	A	A	A<G	G	G	G	G	G	G
	**Vero**	7812	C	U→C	-	U	U	U	U	U	U	U	U	U≈C
	**Vero**	8619	E2	C→U	-	C	C	C	C	C	C	C	C	C≈U
	**Vero**	9855	6K	A→G	-	A	A	A	A	A	A	A	A≈G	A<G
	**Vero**	10419	E1	U→C	-	U	U	U	U≈C	U≈C	U<C	C	C	C
	**Vero**	10509	E1	C→U	-	C	C	C<U	U	U	U	U	U	U
	**Vero**	10542	E1	C→U	-	C	C	C>U	U	U	U	U	U	U
	**Vero**	10896	E1	C→U	-	C	C	C	C	C	C	C	C≈U	C≈U
	**C6/36**	8831	E2	U→C	M→T	U	U	U	U	U	U>C	U≈C	U≈C	U<C
	**C6/36**	9014	E2	A→G	Q→R	A	A	A	A	A	A	A	A≈G	A≈G
	**C6/36**	9305	E2	U→C	I→T	U	U	U	U	U	U>C	U≈C	U≈C	U≈C
	**C6/36**	10670	E1	U→C	V→A	U	U	U>C	U≈C	U<C	U<C	U<C	C	C
	**Vero/C636**	22	5'UTR	A→G	-	A	A<G	G	G	G	G	G	G	G
	**Vero/C636**	66	5'UTR	A→G	-	A	G	G	G	G	G	G	G	G
	**Vero/C636**	742	nsP1	A→U	-	A	A	A	A≈U	U	U	U	U	U
	**Vero/C636**	822	nsP1	C→U	P→L	C	C	C	C≈U	U	U	U	U	U
	**Vero/C636**	3218	nsP2	U→G	S→A	U	U	U	U	U	U	U	U>G	U<G
	**Vero/C636**	5249	nsP3	U→C	W→R	U	U	U	U	U	U	U	U	U<C
	**Vero/C636**	6330	nsP4	U→C	V→A	U	U	U	U	U	U	U	U	U≈C
	**Vero/C636**	6426	nsP4	C→U	A→V	C	C	C	C≈U	U	U	U	U	U
	**Vero/C636**	9591	E2	U→G	-	U	U	U	U≈G	G	G	G	G	G
	**Vero/C636**	10670	E1	U→C	V→A	U	U	U	U	U	U≈C	U<C	U<C	U<C

Table S5: Primers used for the sequencing of CHIKVs.

Some primers were specific to WT or re-encoded viruses as indicated WT or Reenc (final column).

Sequence	Forward/Reverse	nt position	Specificity
ATGGCTGCGTGAGACACA	Forward	1-18	no
GCCAGACACGGAGACGCCAA	Forward	451-470	WT
CCCTGACACGGAGACGCCCA	Forward	451-470	Reenc
CTGTGATACAGTGGTTTCGT	Forward	901-920	WT
CTGTGACACCGTAGTCTCGT	Forward	901-920	Reenc
GCAGAAAACACACACGGTCT	Forward	1351-1370	WT
GCAAAAAACACACACGGTTT	Forward	1351-1370	Reenc
GATTCACGCTTTGGCGGAGC	Forward	1801-1820	no
TGGCAAGTCAGCTATTATCA	Forward	2251-2270	no
TGTAGTGGACACTACAGGCT	Forward	2701-2720	no
GGGGATAAAACTAAATGATA	Forward	3151-3170	no
TGGCTATAACCTTGCACTGC	Forward	3601-3620	no
AGGACAGGTCACCCGAGCAG	Forward	4051-4070	no
CTGCCGCGACAAAGAATGGG	Forward	4501-4520	no
CCCAAAGTACAAAATAGAAG	Forward	4951-4970	no
GGAGACGCGTGACACAGCAA	Forward	5401-5420	no
AAAGCAGCAATCATCCAGAGAC	Forward	5882-5903	no
GCGACAGCATACCTATGTGG	Forward	6620-6639	no
TTCGAGAAGCTCAGAGGGCC	Forward	7451-7470	no
ATTGTATTTTCGAAGTCAAG	Forward	7901-7920	no
GTCTTGCCATCCCAGTTATG	Forward	8351-8370	no
CATCAGCACCGTGTACGATT	Forward	8801-8820	no
ATAACTCCCCTCTGGTCCCG	Forward	9251-9270	no
GGATGTGCATGTGTGCACGA	Forward	9701-9720	WT
GGATGTGCATGTGCGCACGA	Forward	9701-9720	Reenc
CGAGTATAAGACCGTGATAC	Forward	10,140-10,159	Reenc
CCGTCATCCCGTCTCCGTAC	Forward	10,151-10,170	WT

Table S5 (**continued**)**:** Primers used for the sequencing of CHIKVs.

Sequence	Forward/Reverse	nt position	Specificity
CCGTGATACCGAGCCCGTAC	Forward	10,151-10,170	Reenc
TCCAAAGTCGCACACCTGAG	Forward	10,601-10,620	WT
TCCAGTCACGCACCCCTGAA	Forward	10,601-10,620	Reenc
AAATCTCTTTCTCGACGGCC	Forward	11,051-11,070	no
TAAGTATGAAGGTATATGTGTCC	Forward	11,323-11,345	no
GCCATGGCATTGTACATGAACG	Reverse	621-642	WT
GCCATGGCATTATACATGAACG	Reverse	621-642	Reenc
TGCACACCGAGAATGACATT	Reverse	1051-1070	WT
TACATACCGAAAAGGACATC	Reverse	1051-1070	Reenc
TGTATGGGATCAGGTCGGTT	Reverse	1507-1526	WT
AGTATGGTATGAGATCTGTT	Reverse	1507-1526	Reenc
CTCTTTCGTTATACACCATC	Reverse	1951-1970	no
CGACTGGTCTGTTGCATCCA	Reverse	2401-2420	no
CTTTTTGTCTAACTGCGTAA	Reverse	2851-2870	no
TATCCGCGTAATACACAGAC	Reverse	3301-3320	no
GTTGGTAATGGTGTATGCGA	Reverse	3751-3770	no
CACTGTTCTTAAAGGACTCC	Reverse	4201-4220	no
AGTACAGTGCGCCTTCCGTG	Reverse	4651-4670	no
CGCTTAGGTCGAATTGACTA	Reverse	5101-5120	no
CTCCGAAAGTTAGTAGCTCA	Reverse	5551-5570	no
CGGGATTGGACAATCGGACG	Reverse	6001-6020	no
AATCCTCTAACAGCATCAAAGC	Reverse	6824-6845	no
GTCGAGGCTGGTACCTCC	Reverse	7601-7618	no
GGGTATCTGCGCGCATTCAA	Reverse	8051-8070	no
ACATGTTAAGGATGCTTGTA	Reverse	8501-8520	no
GGAATGGAATTTTTCCCGAC	Reverse	8951-8970	no
TGTTGGGTGGTCAGGATACA	Reverse	9401-9420	no
TAGCCAAAACAAAGGTTGCT	Reverse	9851-9870	WT
CAACCAAAACAGAGGTTGTT	Reverse	9851-9870	Reenc
CTCCACGTGTGCTTCGCTCA	Reverse	10,301-10,320	WT
CTCTACATGAGCTTCTGACA	Reverse	10,301-10,320	Reenc
GCAGCCAAATGGTGCTGTGT	Reverse	10,751-10,770	WT
ACAGCCGAATGGGGCGGTAT	Reverse	10,751-10,770	Reenc
AAACTAGGCGCGTCGACCAC	Reverse	10,861-10,880	Reenc
CCATGACATCGCCGTAGCGG	Reverse	11,201-11,220	no
CAATTATGGTATTCATTTGA	Reverse	11,651-11,670	no
TGAAATATTAAAAACAAAATAAC	Reverse	11,789-11,812*	no

*: this primer includes the first base of the polyA tail

Table S6: Primers and probes used for the real time RT-PCR assays.

Some primers and probes were specific to WT or re-encoded viruses as indicated WT or Reenc (final column).

Sequence	Forward/Reverse	nt position	Specificity
TGACCGCCATTGTGTCATCGTTG	Forward	2631-2653	no
CTGGAGACCTCGTGTTAACGTGCTTCAG Probe (5'FAM; 3'TAMRA)	-	2736-2763	no
GACCTCGTATCCACGATAGTCA	Reverse	2788-2809	no
ATGATTCACTTGCGCTTACTGC	Forward	6804-6825	WT
TCCCTGCTGGACTTGATAGAGGC Probe (5'FAM; 3'TAMRA)	-	6860-6882	WT
GAGTTAGGAACATACCTGAT	Reverse	6952-6971	WT
ATGACAGTTTAGCGTTAACAGC	Forward	6804-6825	Reenc
TCCTTACTAGACCTAATAGAAGC Probe (5'FAM; 3'TAMRA)	-	6860-6882	Reenc
ATGTTAAGAACATGCCGCTC	Reverse	6952-6971	Reenc

References

1. Tsetsarkin K, Higgs S, McGee CE, De Lamballerie X, Charrel RN, et al. (2006) Infectious clones of Chikungunya virus (La Reunion isolate) for vector competence studies. Vector Borne Zoonotic Dis 6: 325-337.

2. Xia X, Xie Z (2001) DAMBE: software package for data analysis in molecular biology and evolution. J Hered 92: 371-373.

3. Novembre JA (2002) Accounting for background nucleotide composition when measuring codon usage bias. Mol Biol Evol 19: 1390-1394.
