# Supplementary material for: Random Codon Re-encoding Induces Stable Reduction of Replicative Fitness of Chikungunya Virus in Primate and Mosquito Cells
Source: PLoS Pathog. 2013 Feb 21;9(2):e1003172. doi: 10.1371/journal.ppat.1003172 (PMC3578757; doi:10.1371/journal.ppat.1003172)
Supplement: Text S2 — The re-encoded sequences, the list of the 132 CHIKV sequences extracted from GenBank, the nucleotide sequence of the synthetic RNA transcript used as standard for the universal real time RT-PCR assay and the amino-acid sequence of the recombinant protein used to immunized rabbits. (PDF) [file ppat.1003172.s003.pdf]

SUPPORTING INFORMATION: Text S2

Random codon re-encoding induces stable reduction of replicative fitness of Chikungunya virus in primate and mosquito cells

Antoine Nougairède, Lauriane De Fabritus, Fabien Aubry, Ernest A. Gould, Edward C. Holmes and Xavier De Lamballerie.

Table of Contents

1. Re-encoded sequences 2
2. List of the 132 CHIKV sequences extracted from GenBank 4
3. Nucleotide sequence of the synthetic RNA transcript used as standard for the universal real time RT-PCR assay 4
4. Amino-acid sequence of the recombinant protein used to immunized rabbits 5

1. Re-encoded sequences

Cassette located in the nsP1 Region

Length (nt): 1302

Position (nt) in complete genome: 242-1543

Number of mutations: 264

GATCCTGATTCCACTATTCTAGATATAGGGTCTGCGCCAGCAAGGAGAATGATGTCGGACAGAAAATACCATT
GTGTTTGTCCGATGCGCAGTGCGGAAGACCCTGAGAGACTAGCAAATTATGCGAGAAAGCTAGCCTCCGCC
GCAGGGAAAGTACTGGATAGGAATATCTCTGGGAAAATTGGAGACCTACAAGCAGTGATGGCAGTCCCTGAC
ACGGAGACGCCCACCTTCTGTCTCCACACTGACGTATCTTGCAGGCAAAGAGCTGATGTCGCAATCTACCAA
GATGTTTATGCAGTGCATGCACCCACGTCGTTATACCACCAAGCGATTAAAGGTGTGCGAGTAGCGTACTGG
GTAGGGTTCGATACTACTCCGTTCATGTATAATGCCATGGCGGGGGCATATCCAAGCTACTCGACAAATTGG
GCCGATGAGCAGGTGTTAAAAGCCAAGAATATTGGTCTTTGCAGCACCGATTTGACGGAAGGTAGACGAGGA
AAATTATCTATTATGAGGGGTAAAAAACTTAAACCGTGTGATCGTGTTCTCTTTAGTGTAGGTTCAACGTTGTA
CCCGGAGTCCCGCAAGCTCCTTAAGAGTTGGCATCTGCCCTCGGTGTTCCACTTAAAAGGTAAGCTCTCATT
TACATGTCGCTGTGACACCGTAGTCTCGTGCGAAGGTTATGTTGTCAAAAGGATAACGATGAGCCCTGGCCT
ATACGGAAAAACAACCGGATACGCGGTCACCCACCACGCAGATGGTTTTCTAATGTGCAAGACCACAGATAC
GGTAGACGGAGAGAGGATGTCCTTTTCGGTATGTACTTACGTGCCGGCGACCATTTGCGATCAGATGACCG
GCATATTGGCTACAGAGGTCACGCCGGAGGATGCTCAGAAGTTACTTGTGGGTTTAAATCAAAGGATCGTTG
TAAACGGGAGAACGCAACGGAACACGAATACAATGAAAAATTACTTACTACCAGTGGTCGCTCAAGCATTCTC
CAAGTGGGCAAAAGAGTGTCGGAAAGATATGGAGGACGAAAAATTATTAGGCGTCAGGGAAAGAACACTCAC
CTGTTGCTGCCTCTGGGCTTTCAAGAAGCAAAAAACACACACGGTTTACAAAAGGCCCGACACACAATCCATT
CAGAAAGTACAGGCCGAATTCGATTCCTTCGTTGTGCCGTCATTGTGGTCGTCCGGTTTAAGCATCCCCCTC
AGAACAAGGATTAAGTGGCTTCTCTCCAAGGTTCCAAAAACAGATCTCATACCATACTCCGGGGACGCCCGA
GAG

Cassette located in the nsP4 Region

Length (nt): 1410

Position (nt) in complete genome: 6026-7435

Number of mutations: 298

GCCGTGGCCGCCTGCAATGAGTTCTTGGCAAGGAACTATCCAACAGTGTCCAGTTACCAAATTACTGACGAA
TATGATGCTTATCTAGATATGGTAGACGGGTCGGAATCATGCTTAGATCGAGCGACATTTAATCCGTCTAAGT
TGAGGTCTTATCCGAAACAACATGCATACCACGCGCCTAGCATAAGAAGCGCTGTACCGAGTCCTTTCCAGA
ATACTCTGCAGAACGTTTTAGCCGCCGCCACGAAAAGGAACTGCAATGTTACACAGATGAGGGAACTCCCAA
CATTAGACTCCGCTGTATTTAACGTCGAATGTTTCAAGAAGTTTGCATGCAATCAAGAGTATTGGGAAGAATTT
GCCGCCTCCCCTATCAGAATAACCACAGAAAATTTAGCCACTTACGTCACTAAATTAAAAGGCCCTAAGGCCG
CCGCGCTGTTTGCTAAAACTCATAATTTGCTTCCCCTGCAGGAAGTTCCCATGGATAGGTTTACTGTCGATAT
GAAAAGGGATGTAAAAGTGACTCCAGGTACCAAACATACTGAAGAAAGGCCAAAGGTGCAAGTGATCCAAGC
GGCAGAGCCCTTAGCGACAGCCTACTTATGTGGAATTCATAGAGAATTGGTGAGAAGATTAAACGCAGTCCT
CTTGCCTAACGTGCATACTCTTTTCGACATGTCCGCTGAAGACTTCGATGCCATAATAGCAGCTCATTTTAAA
CCCGGAGATACTGTGTTGGAAACGGATATTGCATCATTCGACAAAAGCCAAGATGACAGTTTAGCGTTAACA

GCTTTGATGTTGTTGGAAGACTTAGGGGTCGATCACTCCTTACTAGACCTAATAGAAGCTGCCTTCGGGGAG
ATATCATCTTGCCATCTACCGACAGGTACGCGCTTTAAATTTGGCGCCATGATGAAGAGCGGCATGTTCTTAA
CATTATTCGTAAATACCTTGTTAAACATTACCATCGCAAGTCGAGTGCTAGAGGACCGTTTAACTAAGAGTGC
GTGTGCGGCATTTATAGGTGACGATAACATAATCCATGGGGTTGTGAGTGATGAATTAATGGCTGCCAGGTG
TGCTACCTGGATGAACATGGAGGTGAAAATAATCGATGCAGTGGTCTCCCTGAAGGCCCCATACTTCTGCGG
GGGTTTTATCCTCCATGACACTGTTACCGGCACCGCATGTAGAGTAGCTGACCCGTTAAAAAGGTTGTTTAAA
CTCGGCAAGCCGCTAGCGGCTGGGGATGAACAGGACGAGGATAGGAGGCGAGCGTTAGCAGATGAGGTGA
TCAGGTGGCAACGAACTGGATTGATCGACGAGCTAGAGAAAGCGGTTTACTCAAGATACGAGGTGCAGGGG
ATTAGTGTGGTTGTGATGTCAATGGCCACTTTCGCC

Cassette located in the E1/E2 Region

Length (nt): 1500

Position (nt) in complete genome: 9523-11022

Number of mutations: 320

GTTACGTGGGGAAATAATGAGCCGTACAAATATTGGCCGCAGTTGTCCACCAACGGTACAGCTCATGGTCAT
CCGCATGAGATTATACTCTACTATTATGAACTATATCCAACAATGACTGTCGTTGTCGTTAGTGTGGCAACGTT
CATACTACTTTCGATGGTAGGTATGGCTGCGGGGATGTGCATGTGCGCACGACGCAGATGCATAACTCCGTA
TGAATTAACTCCCGGCGCAACAGTACCCTTCCTACTAAGCTTAATCTGCTGCATCAGGACAGCAAAGGCGGC
CACTTACCAGGAGGCCGCGATTTACCTGTGGAATGAACAACAACCTCTGTTTTGGTTGCAAGCATTAATCCCG
CTAGCAGCTCTCATAGTTCTGTGTAACTGTCTGAGGTTACTCCCCTGCTGCTGCAAAACGTTGGCATTCCTAG
CAGTGATGTCCGTGGGTGCCCACACTGTGTCAGCGTATGAACACGTGACTGTCATTCCGAATACGGTCGGG
GTGCCGTATAAAACCTTGGTGAATAGACCCGGATACTCTCCCATGGTTCTGGAAATGGAGCTGTTGAGCGTA
ACTCTGGAGCCTACTCTCTCGCTGGATTACATCACGTGCGAGTATAAGACCGTGATACCGAGCCCGTACGTG
AAGTGTTGTGGAACTGCAGAGTGCAAAGATAAGAACTTGCCCGACTACTCTTGTAAAGTTTTCACAGGCGTTT
ACCCCTTCATGTGGGGTGGGGCATACTGTTTTTGTGACGCAGAAAATACGCAGTTGTCAGAAGCTCATGTAG
AGAAGAGCGAGAGCTGTAAAACAGAGTTCGCTTCCGCATACAGAGCCCATACTGCATCCGCTTCCGCAAAGC
TACGCGTTCTATATCAAGGCAATAATATTACTGTGACCGCCTACGCCAACGGAGATCATGCAGTTACTGTTAA
AGACGCAAAATTCATTGTAGGGCCTATGAGCAGCGCATGGACCCCCTTTGACAACAAGATAGTGGTTTATAAA
GGAGACGTATACAATATGGATTATCCGCCCTTTGGTGCTGGCAGACCTGGGCAATTTGGTGATATCCAGTCA
CGCACCCCTGAAAGTAAGGATGTTTACGCTAATACACAGCTCGTTCTCCAGAGGCCGGCAGTAGGCACGGTA
CATGTACCCTACTCACAGGCCCCTTCTGGTTTTAAATACTGGCTGAAGGAACGCGGTGCGTCGCTTCAACAT
ACCGCCCCATTCGGCTGTCAAATTGCCACAAACCCGGTTAGGGCGGTCAACTGCGCTGTAGGAAACATGCC
TATTAGTATCGATATCCCGGAGGCGGCATTCACCAGGGTGGTCGACGCGCCTAGTTTAACGGATATGTCGTG
CGAAGTCCCTGCTTGTACTCACAGTAGTGATTTTGGTGGCGTAGCAATCATAAAGTACGCTGCCTCCAAAAAA
GGAAAATGTGCGGTGCACTCGATGACAAATGCAGTTACTATTCGGGAAGCCGAG

2. List of the 132 CHIKV sequences extracted from GenBank

GenBank accession numbers: HM045818, HM045819, HM045820, AY726732, HM045786, HM045807, HM045785, HM045798, HM045804, HM045816, HM045815, HM045817, HM045810, HM045787, HM045796, HM045802, HM045789, HM045790, HM045800, HM045791, L37661, EU703759, HM045797, HM045808, HM045814, EF027141, HM045788, HM045803, HM045813, EF027140, EF452494, HM045805, HM045811, HM045792, HM045809, EF027139, HM045812, HM045784, HM045823, HM045793, HM045822, HM045821, AF369024, NC_004162, AF490259, EF452493, EU703760, EU703761, EU703762, HM045795, AB455493, AB455494, EF027138, FJ000069, GQ428212, GQ428214, EF210157, FJ445427, FJ445484, FJ445502, FJ445431, FJ445432, FJ445430, FJ445445, FJ445433, FJ445463, FJ445443, GU199352, GU199353, GQ428213, EF027137, HM045801, FJ513628, FJ513679, GU013528, FJ513657, HM045799, FJ513635, FJ513637, GU013529, FJ000066, FJ000063, FJ000064, GQ428211, FJ000062, FJ000068, FJ000065, EF027136, HM045794, FJ445510, GQ428210, EF027134, FJ513654, EF027135, FJ000067, GQ428215, GU199351, FJ445428, FJ807899, GU199350, GU189061, EU244823, GU013530, FJ513629, FJ807896, FJ445511, EU564335, EU372006, FJ807898, FJ445426, FJ513632, FJ513645, FJ513675, FJ513673, AM258990, AM258991, AM258993, AM258994, EF012359, EU037962, FJ959103, AM258992, FU759460, GM839372, FU759461, GM839373, FU759462, GM839374, FU759463, GM839375, FU759464, GM839376.

3. Nucleotide sequence of the synthetic RNA transcript used as standard for the universal real time RT-PCR assay

Here is represented the dsDNA sequence which was synthesized (Eurogentec). This synthetic oligonucleotide, that contains a T7 RNA polymerase promoter site (underline) was then used as template for *in vitro* transcription (MEGAshortscript, Ambion). The first base of the RNA transcript is in bold. Purified (MEGAclear, Ambion) *in vitro* transcription products were used as standard for the universal real time RT-PCR assay.

<u>TAATACGACTCACTATA**G**GGAGA</u>CACTGCCTGTGACCGCCATTGTGTCATCGTTGCATTACGAAGGCAAAAT
GCGCACTAATTATAGCGGCCGCTTATTAATTGTAGTGGACACTACAGGCTCAACAAAACCTGACCCTGGAGA
CCTCGTGTTAACGTGCTTCAGAGGGTGGGTTAAACAACTGCAAATTGACTATCGTGGATACGAGGTCATGAC
AGCAG

4. Amino-acid sequence of the recombinant protein used to immunize rabbits

NKQICVTTRRIEDFNPTTNIIPANRRLPHSLVAEHRPVKGERMEWLVNKINGHHVLLVSGCSLALPTKR
VTWVAPLGVRGADYTYNLELGLPATLGRYDLVVINIHTPFRIHHYQQCVDHAMKLQMLGGDSLRLLKP
GGSLLIRAYGYADRTSERVICVLGRKFRSSRALKPPCVTSNTEMFFLFSNFDNGRRNFTTHVMNNQLN
AAFVGQATRAGCAPSYRVKRMDIAKNDEECVVNAANPRGLPGDGVCKAVYKKWPESFKNSATPVGT
AKTVMCGTYPVIHAVGPNFSNYSESEGDRELAAAYREVAKEVTRLGVNSVAIPLLSTGVYSGGKDRLT
QSLNHLFTAMDSTDADVVIYCRDKEWEKKISEAIQMRT
